# *In Silico* Discovery and Characterization of a Novel Nuclear Transcription Factor‑Y (NF-Y) Inhibitor with Antimitogenic Properties

**DOI:** 10.1021/acs.jmedchem.5c03508

**Published:** 2026-03-20

**Authors:** Reza Ebrahimighaei, Jon Lees, Robin A. Corey, Boyi Xiao, Christopher Williams, Himali Y. Godage, Vealmurugan Sekar, Hunaid Vohra, Deborah Shoemark, Andrew Newby, Mark Bond

**Affiliations:** † Translational Health Sciences Bristol Medical School, University of Bristol, Bristol BS2 8HW, U.K.; ‡ School of Physiology, Pharmacology and Neuroscience, Biomedical Sciences Building, 1980 University of Bristol, Bristol BS8 1TD, U.K.; § School of Chemistry, Faculty of Science, 1980 University of Bristol, Cantock’s Close, Bristol BS8 1TS, U.K.; ∥ Bristol Heart Institute, University Hospitals Bristol, NHS Foundation Trust, Bristol BS1 3NU, U.K.

## Abstract

Nuclear Transcription Factor-Y (NF-Y) is a transcription factor that binds CCAAT motifs to regulate gene expression, controlling cell proliferation, metabolism, and differentiation. NF-Y dysregulation contributes to diverse pathologies, including cancer, neurological disorders, cardiovascular disease, and tissue fibrosis. Using *in silico* molecular docking, we screened a library of eight million compounds to identify molecules targeting a pocket on the NF-YB/NF-YC dimer. We identified one compound, designated NFYi5, that was able to reduce the NF-Y activity. NFYi5 reduced mRNA levels of NF-Y target genes, while sparing housekeeping gene expression, and inhibiting cell proliferation. Mechanistic studies revealed that NFYi5 impaired NF-Y–DNA binding and accelerated NF-YA protein degradation, reducing its half-life from 16.5 ± 1.5 h to 8.5 ± 0.7 h. Together, these data establish NFYi5 as a small-molecule that can reduce NF-Y activity and is associated with antimitogenic properties. This proof-of-concept study demonstrates that NF-Y is pharmacologically tractable and highlights NFYi5 as a potential lead compound for therapeutic development in NF-Y-driven diseases.

## Graphical abstract

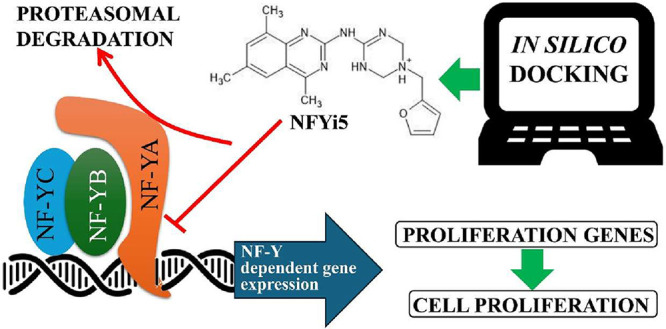

## Introduction

Nuclear Transcription Factor Y (NF-Y) is a heterotrimeric transcription factor, composed of NF-YA, NF-YB, and NF-YC subunits, which binds to the CCAAT DNA sequence in the promoter regions of target genes to either activate or repress their transcription.^1^ Although early studies reported that the CCAAT motif is present in the proximal promoters of many mammalian genes and involved in regulating their basal expression,^1^ recent studies have highlighted a specific role for NF-Y binding to the CCAAT motif in driving specific gene expression programmes in response to a diverse physiological signals, including responses to mitogens, TGF-β,^2^ mechanical signals,^3^ and interferon-gamma.^4^

NF-Y-dependent gene transcription has been implicated in numerous physiological processes, including the regulation of metabolism,^5^ cell differentiation^6,7^ and cell cycle progression.^8^ Importantly, dysregulation of NF-Y has also been implicated in several pathological processes, including cancer,^9^ tissue fibrosis,^3^ neurological disorders,^7^ and cardiovascular disease,^10^ where NF-Y drives uncontrolled proliferation,^8^ metabolic reprogramming,^11^ resistance to apoptosis, and aberrant gene expression.^11^ This suggests that targeting the NF-Y function may be beneficial in these disorders.

The NF-Y subunits NF-YB and NF-YC dimerize via a conserved histone fold domain (HFD) that has similarities to the HFDs of histones H2B/H2A.^12^ This NF-YB/NF-YC dimer can bind to DNA in a nonsequence-selective manner. Selectivity of CCAAT sequences is conferred by the NF-YA subunit, which contains a DNA-binding domain that interacts with the DNA minor groove at CCAAT sequences, inducing a 80° bend in the DNA helix.^13^ The NF-YA subunit has been suggested to act as the main regulator of NF-Y activity due to its role in directing high-affinity, sequence-specific DNA binding and the modulation of NF-YA protein levels in response to various cellular signals.^14^ For example, NF-YA expression in fibroblasts is elevated in response to TGF-β stimulation or in response to serum mitogens, where it promotes collagen expression and fibroblast proliferation.^15^ NF-YA levels are downregulated during muscle cell differentiation, where forced NF-YA expression delays early muscle cell differentiation.^16^ NF-YA levels are modulated during cell-cycle phase transition, increasing through G1-S and decreasing in G2-M.^17^ Furthermore, NF-YA levels are also elevated during pathological conditions, including breast cancer,^18^ renal cell carcinoma,^19^ and in response to vascular injury.^10^

We recently demonstrated a major role for the transcription NF-Y as a mechano-sensitive regulator of cardiac fibroblast proliferation.^8^ We showed that cardiac fibroblasts interacting with a stiff extracellular matrix (ECM) expressed higher levels of NF-YA protein and had elevated levels of NF-Y activity compared to cells interacting with a soft ECM. This stiffness-dependent upregulation of NF-YA was responsible for stiffness-dependent cell proliferation. This suggests that the pharmacological inhibition of NF-Y activity may be a viable way to limit the cellular response to increased ECM stiffness and possibly limit fibrosis. Therapeutic targeting of NF-Y may also be beneficial in other NF-Y-driven pathologies, including cancer, cardiovascular, and neurological diseases.

To date, only one study has reported the identification of a small molecule inhibitor specifically designed to inhibit NF-Y.^20^ Nardone et al. described the inhibition of NF-Y DNA binding by Suramin, which was identified from an *in-silico* screen of 1280 compounds for their ability to bind the NF-Y histone fold domain (HFD). However, due to its large, flexible, and multifunctional nature, suramin tends to be a nonselective drug. This may in part be due to similarities between the NF-Y HFD and other HFD-containing proteins, which could also interact with suramin. Moreover, it is not known if suramin can inhibit NF-Y activity in living cells. Hence, there is a need for a selective NF-Y inhibitor that acts independently of the NF-Y HFD. Here, we used the Bristol University Docking Engine (BUDE)^21^ to screen a virtual library of more than eight million drug-like compounds to identify novel NF-Y inhibitory compounds. We describe a small molecule with NF-Y inhibitory properties. This molecule displays antimitogenic effects in cardiac fibroblasts, consistent with the role of NF-Y in cell-cycle regulation. We present data that demonstrate that this molecule reduces NF-Y DNA binding as well as induces NF-YA degradation. We provide proof of concept that NF-Y is amenable to pharmacological inhibition, and our findings describing the NFYi5 inhibitor lay the groundwork for future therapeutic strategies to target NF-Y-driven pathologies.

## Results

### *In S* *ilico* Docking

We examined the crystal structure of the NF-Y: DNA complex (4AWL.pdb^13^) to identify potential pockets that, when targeted by small drug-like compounds, may result in disruption of NF-Y function or activity. We identified a region on the NF-YB/NF-YC dimer (bordered by residues Lys78, Glu52, Glu82, and Glu86 in NF-YB) that accommodates the side chain of Arginine 266 of NF-YA (Figure 1A–C). Arginine-266 of NF-YA displays a high degree of cross-species conservation (Figure 1D), suggesting that it plays a functionally important role. NF-YA Arginine 266, located in the A1–A2 linker region of NF-YA between the N-terminal α-helix that interacts with the NF-YB/NF-YC dimer, and the C-terminal DNA binding α-helix, that confers CCAAT DNA binding specificity. We speculated that docking of the NF-YA Arginine-266 side chain into this pocket may play a role in determining the correct positioning of the DNA binding domain, and hence NF-Y DNA binding. We therefore performed molecular docking using BUDE, with the search area defined as a 15 × 15 × 15 Å^3^ grid centered on the zeta carbon atom of the NF-YA Arginine 266 residue (Figure 1C and Supplement Figure 1).

**Figure 1:**
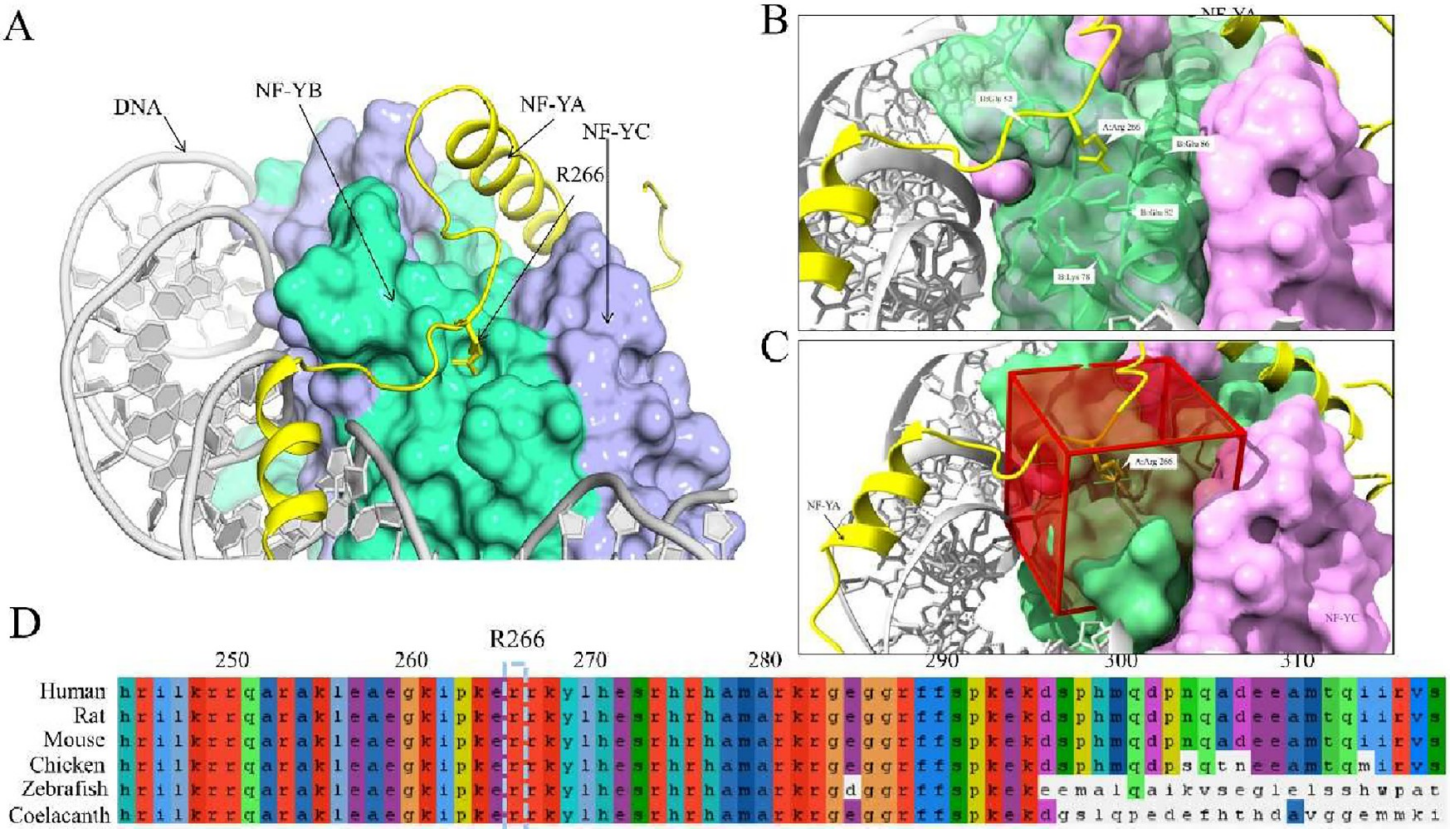
Pocket on NF-YB accommodates the cross-species conserved Arginine 266 of NF-YA. Render of the NF-Y heterotrimer (4AWL.pdb) showing the pocket on NF-YB that was targeted for docking in BUDE (A). Enlarged view of the pocket (B). Red boxes indicating the 15 × 15 × 15 Å BUDE search are (C). Alignment of part of the NF-YA protein sequence from Human, Rat, Mouse, Chicken, Zebrafish, and Coelacanth. The conserved Arginine-266 residue is boxed (D).

A library of >8 million compounds, obtained from the clean, druglike subset of the ZINC8 database,^22,30^ was used for docking studies. Approximately 20 conformers per compound were generated using Confort^23^ (Certara Inc.), resulting in a library of approximately 160 million distinct structures that were docked into the NF-YB/NF-YC pocket. The BUDE docking produced a ranked list of conformers from 160 million. The best scoring conformers were manually curated into a final list of 7 compounds for testing in vitro used the following criteria: (i) visual inspection to identify compounds that interacted with NF-YB/NF-YC pocket that accommodates the NF-YA R266 side chain (ii) maximizing the chemical diversity of the initial test set; (iii) favorable calculated (*c*Log*P*) or experimental (log *P*) solubility; and (iv) actual compound availability for purchase at a reasonable (<£200) cost per screening sample.

### NFYi5 Inhibits NF-Y Activity

The shortlisted set of 7 compounds (Figure 2 and Supplement Table 2) was first assayed for their ability to inhibit NF-Y-dependent transcriptional activity in cardiac fibroblasts that had been transiently transfected with a secreted bioluminescent nanoluciferase (sNLUC) reporter gene enzyme, which is expressed under the control of a synthetic promoter region containing five copies of a consensus NF-Y (CCAAT) DNA-binding element. The reporter construct was validated by showing that the expression of secreted nanoluciferase (sNLUC) enzyme activity was significantly stimulated by overexpression of NF-YA (Supplement Figure 2). Furthermore, we previously reported that this reporter construct is inhibited by siRNA-mediated silencing of NF-YA or by expression of a dominant negative mutant of NF-YA.^8^ Therefore, this reporter plasmid faithfully reports NF-Y-dependent transcriptional activity. Only one of the seven compounds shortlisted (NFYi5) significantly (>60%) inhibited NF-Y-sNLUC reporter activity, without significantly affecting cell viability (Figure 3), indicating that this compound inhibited NF-Y-dependent transcriptional activity. Importantly, the addition of compound to cell-free media containing sNLUC enzyme did not result in a loss of enzyme activity (Figure 3C), indicating that the reduction of activity observed in the cell-based experiments is not simply due to poisoning of the NLUC enzymatic reaction.

**Figure 2:**
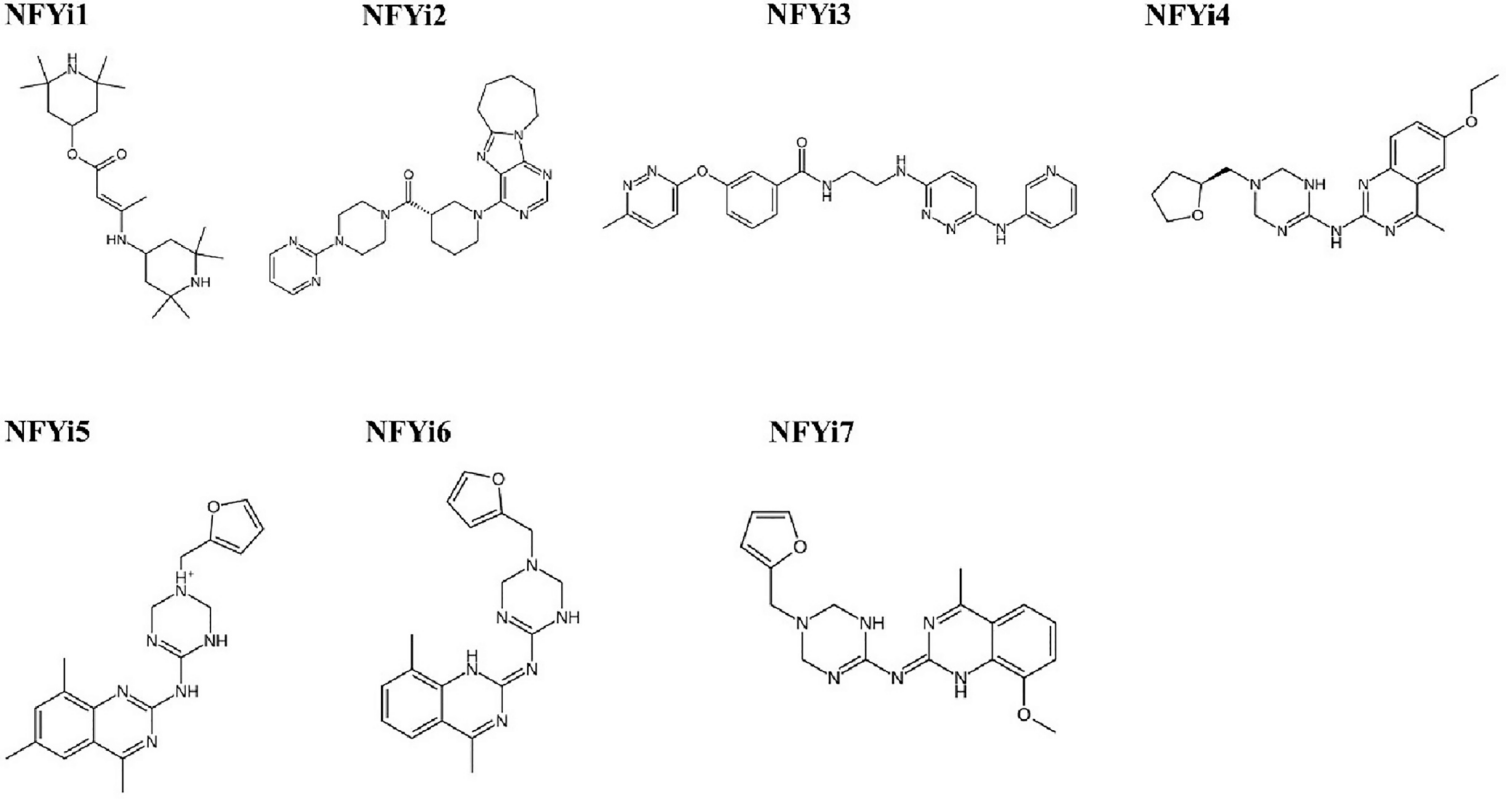
Structures of shortlisted compounds tested for NF-Y inhibitory properties.

**Figure 3:**
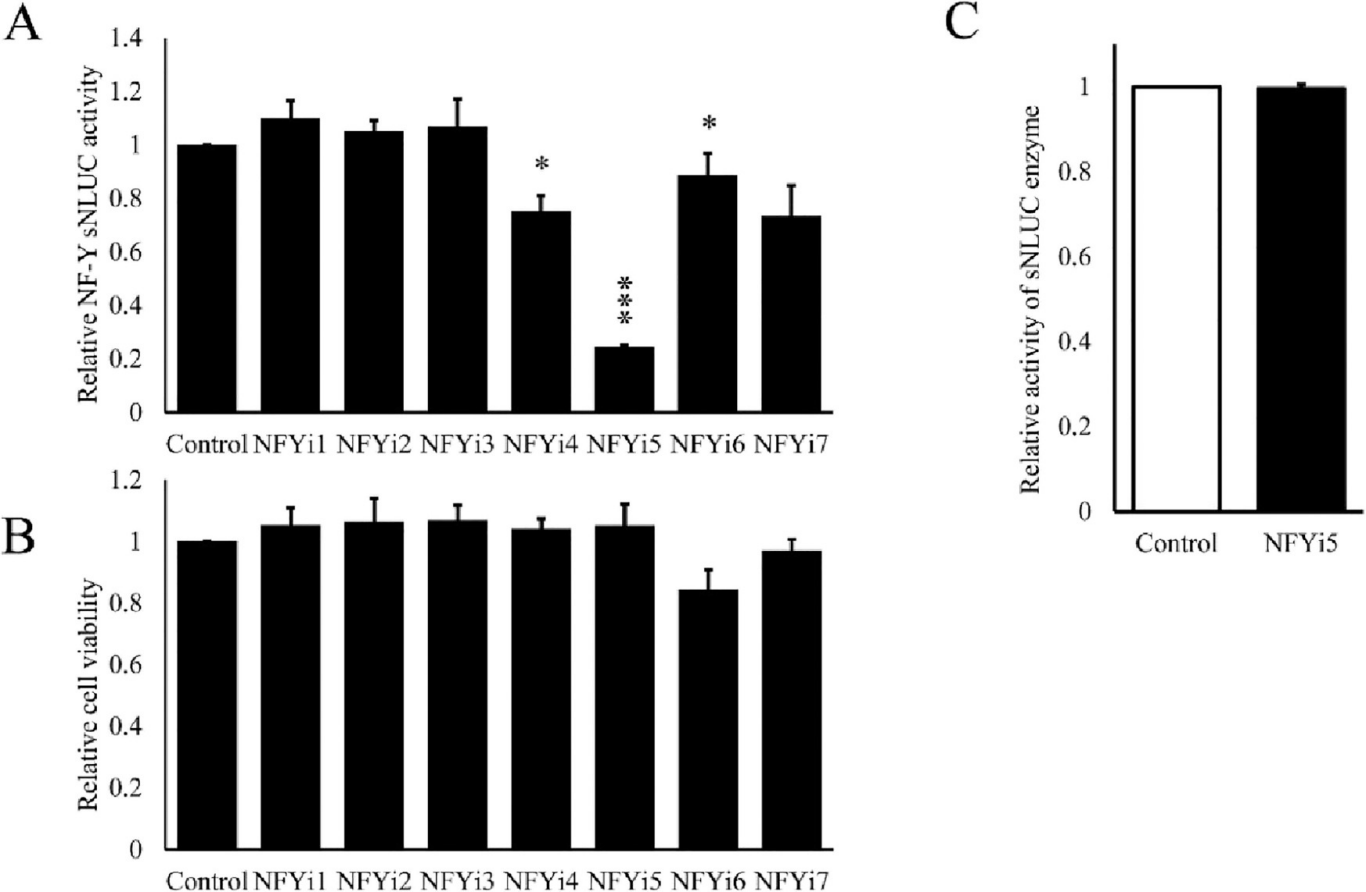
NFYi5 inhibits NF-Y activity without affecting cell viability. Cells were transiently transfected with pNL2.3-NF-Y-sNLUC before being treated with 30 μM of each compound for 18 h. Media was replaced and media conditioned for a further 4 h. Secreted NLUC activity was quantified (A; *n* = 3). Cell viability was quantified using PrestoBlue assay (B; *n* = 3). Media containing the NLUC enzyme was assayed with or without the addition of NFYi5 (30 μM), in the absence of cells, to test if NFYi5 was poisoning the NLUC activity assay (C; *n* = 3).

The BUDE-generated docking pose for NFYi5 (Figure 4) shows the compound occupying the pocket on NF-YB (created mainly by amino acids Lys78, Glu52, Glu82, and Glu86), with the central triazine ring occupying the space that normally accommodates the Arginine-266 side chain of NF-YA. Since BUDE only uses static models for docking, we sought to test if NFYi5 docking to NF-Y is stable over time, and refine the initial pose, by performing molecular dynamics simulations (Supplementary Video 1). First, we evaluated the initial docking site on the NF-YB/NF-YC–DNA interface by running five independent MD replicates. One of these trajectories remained confined near the pocket, exhibiting a low and steady ligand RMSD around 0.4 nm (Figure 5A and Supplement Figure 3). To test whether the stable behavior reflects energetic consistency, we computed an MM/PBSA time series (Figure 5B). The enthalpic component remained consistently negative with a stable moving average, supporting a bound state with favorable protein–ligand interactions at this site. Based on the most stable frame from this simulation, we refined the inhibitor conformation and performed a redocking with GNINA (Figure 5C). Compared to the original BUDE pose, the redocked pose realigns the aromatic core deeper into the microgroove, restores hotspot contacts at the protein–DNA interface, and eliminates clashes observed in the unstable replicates.

**Figure 4:**
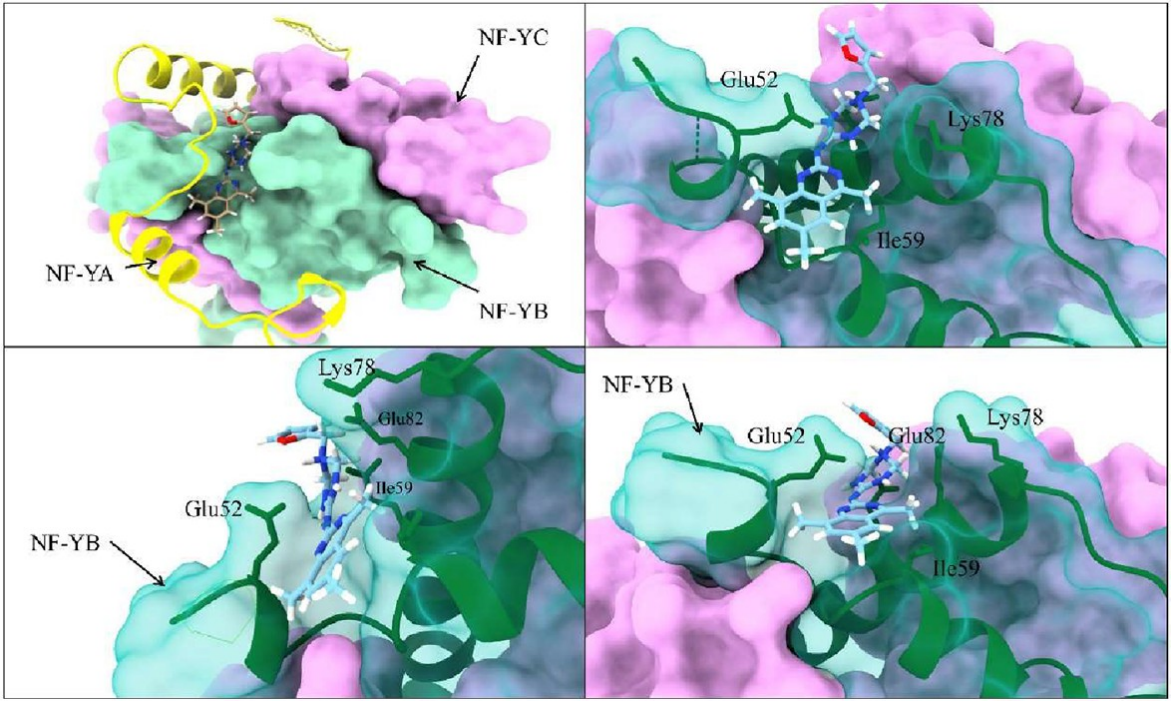
BUDE docking pose for NFYi5 with NF-Y. Docking pose of NFYi5 with the NF-Y heterotrimer (4AWL.pdb) predicted by BUDE. NF-YA in yellow, NF-YB in green, and NF-YC in pink.

**Figure 5:**
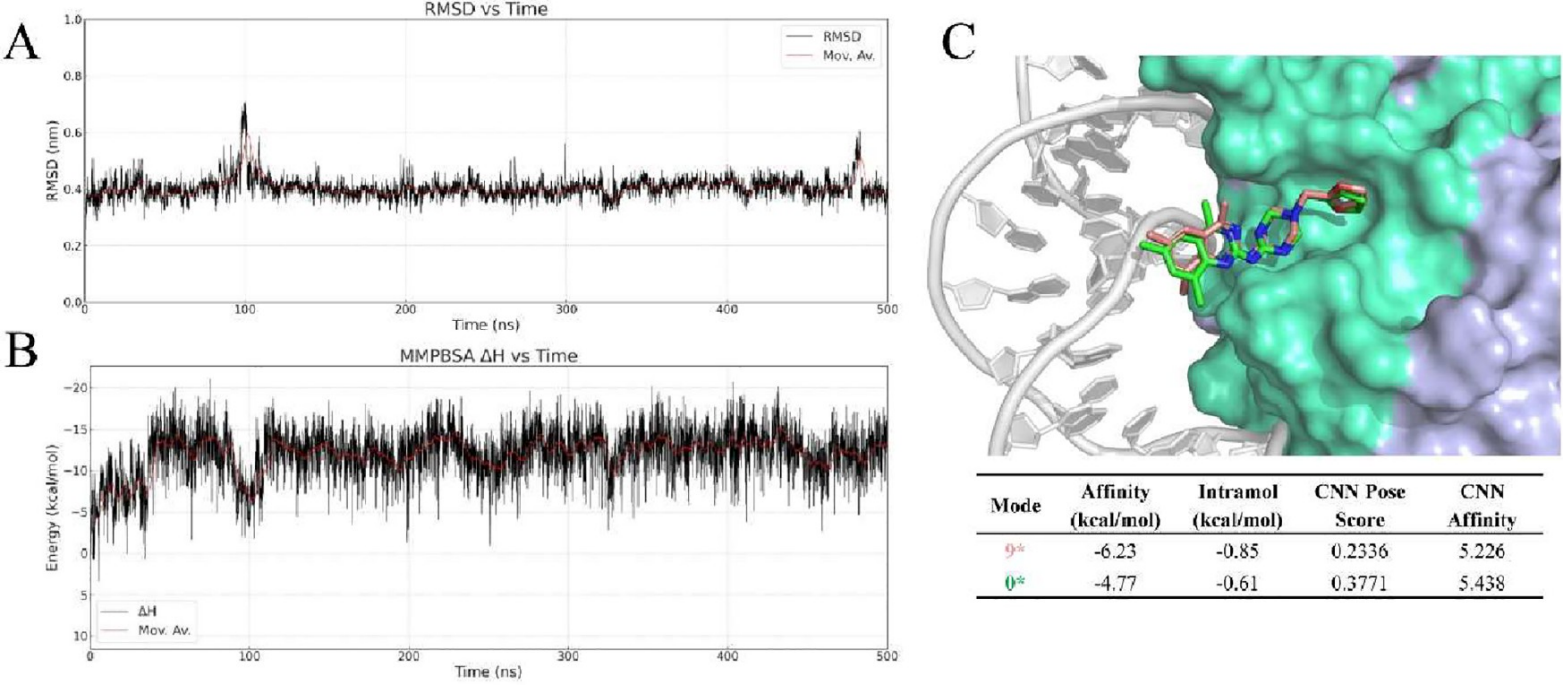
Molecular Dynamics Simulation and redock**.** Ligand RMSD at the initial docking site (A). MM/PBSA enthalpy time series for the bound complex (B). GNINA redocking into the MD-refined pocket of NF-Y (4AWL.pdb). Original Pose (Green); Redock Pose (Pink) (C).

We overlaid the refined NFYi5 pose at NF-YB onto the original NF-Y: DNA complex. To our surprise, both NFYi5 and Arg266 can fit into the NF-YB pocket simultaneously. We subjected the resultant complex to additional MD analysis and analyzed the dynamics of NF-YA in both the presence and absence of the inhibitor compound. Figure 6 shows the residues involved in the primary interactions of NFYi5. Root-mean-square fluctuation (RMSF) analysis showed that the inhibitor reduced the dynamic flexibility of NF-YA substantially, particularly in the linker region (residues ∼250–270) (Figure 6), where RMSF values decreased from 0.8–0.9 nm to 0.3–0.4 nm upon ligand binding. This region encompasses the lysine cluster and the R266 residue that interacts directly with the NF-YB binding pocket.

**Figure 6:**
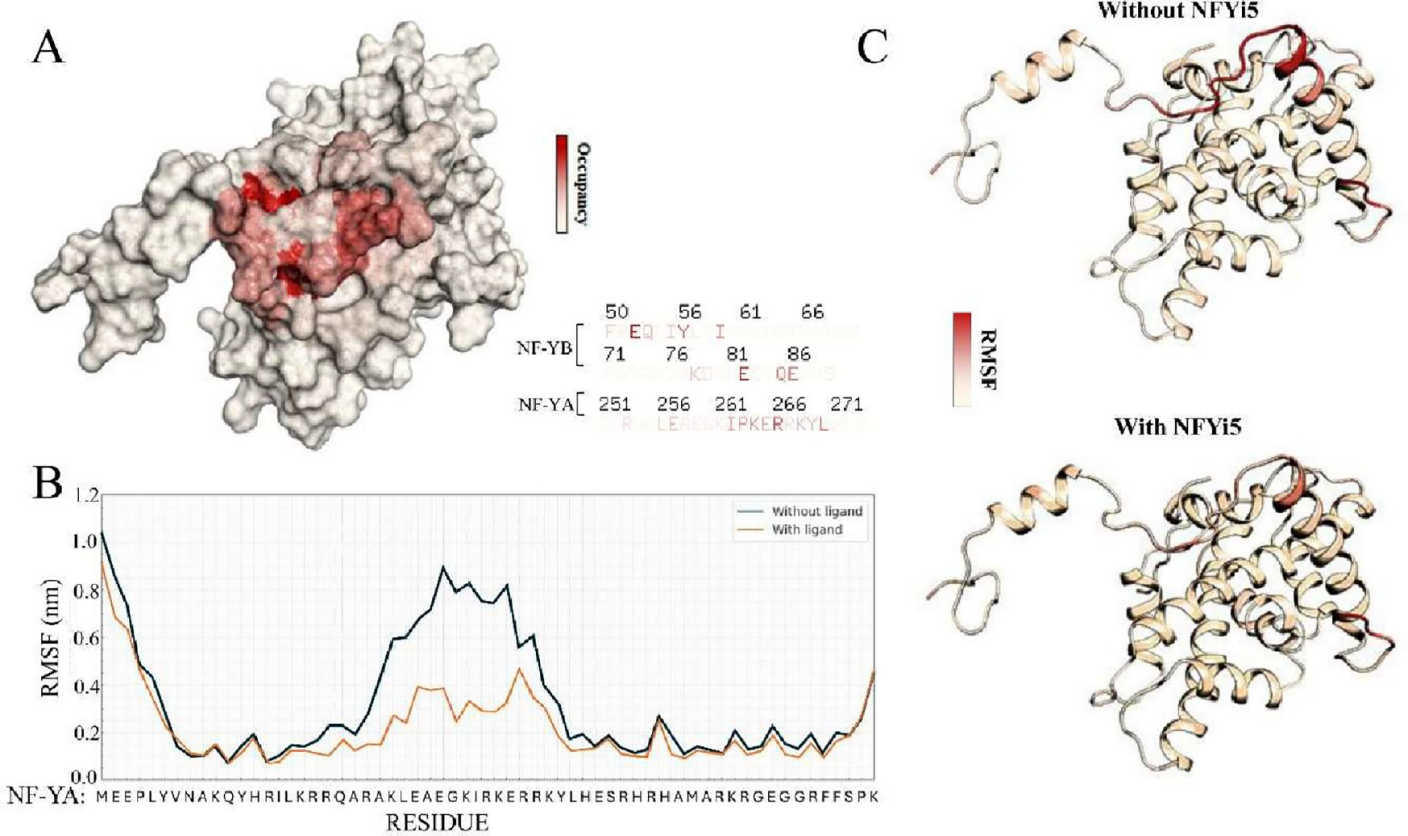
Inhibitor binding induces conformational changes in NF-YA structure and dynamics**.** Molecular dynamics simulation of NF-YA/NY-YB/NF-YC in the presence or absence of NFYi5. (A) Heatmap render of NF-YA/NF-YB/NF-C showing interactions made between NFYi5 and the protein (occupancy), with red regions exhibiting a higher contact likelihood. (B) RMSF of NF-YA residues in the presence (orange line) or absence (blue line) of NFYi5. (C) Heatmap showing RMSF of NF-YA/NY-YB/NF-YC in the presence or absence of NFYi5. Red regions have higher degrees of flexibility.

We next compared the effect of different concentrations of NFYi5 on the activity of reporters under the control of promoters containing or lacking NF-Y binding elements. Treatment with increasing concentrations of NFYi5 dose dependently inhibited activity of the NF-Y reporter in both human and rat cardiac fibroblasts (Figure 7A–C), further confirming our initial finding. In human cells, we calculated the NFYi5 IC_50_ at 19.95 μM. In rat cells, the IC_50_ was calculated as 12.73 μM. Interestingly, these values were close (within 2–3 fold) to the calculated affinity of NFYi5 for NF-YB/NF-YC (CNN Affinity of 5.226, p*K* ∼ 6 μM; Figure 5C). Importantly, the activity of reporters under the control of the CMV or UBC promoters was not significantly affected by either dose of NFYi5 (Figure 7D,E). Activity of a reporter regulated by NF-κB displayed a modest stimulation at the higher dose of NFYi5 (Figure 7F). These data indicate that treatment of cells with NFYi5 is associated with inhibition of NF-Y activity, with at least some selectivity, based on the small number of NF-Y independent promoters tested.

**Figure 7:**
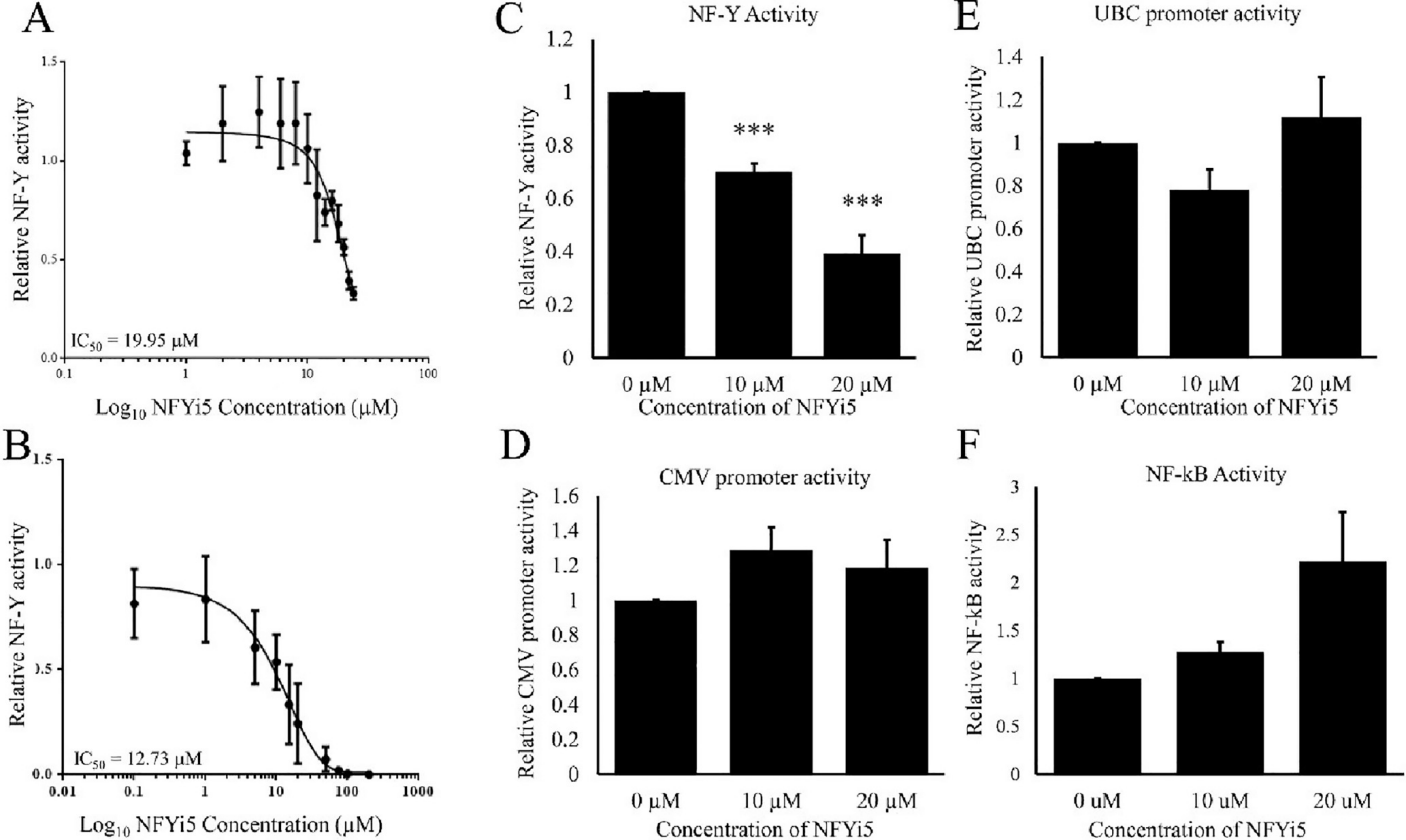
NFYi5 preferentially inhibits NF-Y reporter gene activity. Human (A) and Rat (B and C) cardiac fibroblasts were transiently transfected with pNL2.3-NF-Y-sNLUC and treated with the indicated doses of NFYi5 for 18 h. Secreted NLUC activity was then assayed in 4-h conditioned media (A; *n* = 4). Rat cardiac fibroblasts were transiently transfected with reporter plasmids that report NF-Y (B; *n* = 4), CMV (D; *n* = 4), UBC (E; *n* = 4) or NF-κB (E; *n* = 4) activity. Cells were treated with the indicated concentrations of NFYi5 for 18 h. Media was replaced and conditioned for a further 4 h and reporter activity quantified. ** indicates *p*, 0.01. *** indicates *p* < 0.001. one-way ANOVA with Student–Newman–Keuls post-test.

### NFYi5 Inhibits NF-Y Target Gene Expression

We analyzed the effect of NFYi5 on the mRNA levels of multiple NF-Y-target genes and NF-Y-independent housekeeping genes to further test if NFYi5 can selectively inhibit NF-Y-dependent gene expression. Treatment of human cardiac fibroblast cells with NFYi5 dose dependently reduced the mRNA levels of the NF-Y-target genes (CCNA2, CCNB2 ^31^) without reducing the levels of NF-Y-independent housekeeping gene GAPDH (Figure 8A). We previously demonstrated NF-Y-dependent regulation of CCNA2, CCNB1, ECT2, and DDX11 in rat cardiac fibroblasts.^8^ NFYi5 also significantly repressed the mRNA levels of these NF-Y target genes (CCNA2, CCNB1, ECT2, and DDX11) in rat CF without affecting the levels of multiple housekeeper gene mRNAs (TBP, 36B4, GAPDH, and UBC; Figure 8B,C), further suggesting that NFYi5 inhibits NF-Y-dependent gene expression, with at least some degree of selectivity.

**Figure 8:**
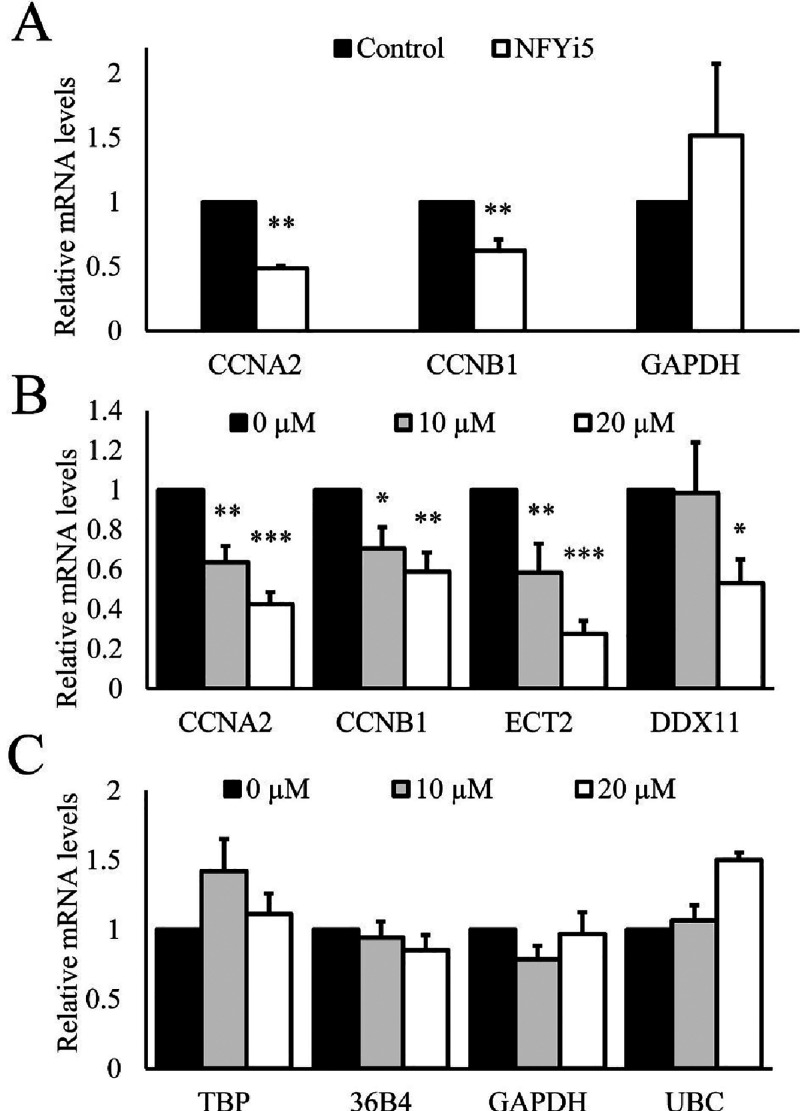
NFYi5 preferentially inhibits the mRNA levels of NF-Y target genes. Human (A; *n* = 3) and Rat (B and C; *n*-4) cardiac fibroblasts were treated with the indicated concentrations (20 μM in A) of NFYi5 for 18 h. Total RNA was analyzed by RT-qPCR for the NF-Y-target gene mRNAs, CCNA1, CCNB1, ECT2 and DDX11 and the NF-Y-independent gene mRNAs TBP, 36B4, GAPDH and UBC. * Indicates *p* < 0.05, ** indicates *p* < 0.01. *** indicates *p* < 0.001. One-way ANOVA with Student–Newman–Keuls post-test.

### NFYi5 Inhibits Cardiac Fibroblast Proliferation

We and others have previously shown that NF-Y is required for cell proliferation.^8,32^ We therefore quantified the effect of NFYi5 on the proliferation of rat and human cardiac fibroblasts. Treatment of cells with NFYi5 dose-dependently inhibited incorporation of Edu into both rat and human cells (Figure 9A,B). Proliferation of rat cells was significantly inhibited with 15 μM NFYi5 (Figure 9A). Proliferation of human cells was significantly inhibited by 5 μM NFYi5, with maximal effects at 20 μM (Figure 9B). Consistent with a reduction in Edu incorporation (indicated by reduced S-phase progression), we also observed a significant reduction in cell number after 24 and 48 h with 20 μM NFYi5 (Figure 9C,D). The concentrations of NFYi5 effective at inhibiting the proliferation of human and rat cells are consistent with the calculated IC_50_ values of 19.95 and 12.73 μM, respectively, for the inhibition of NF-Y activity.

**Figure 9:**
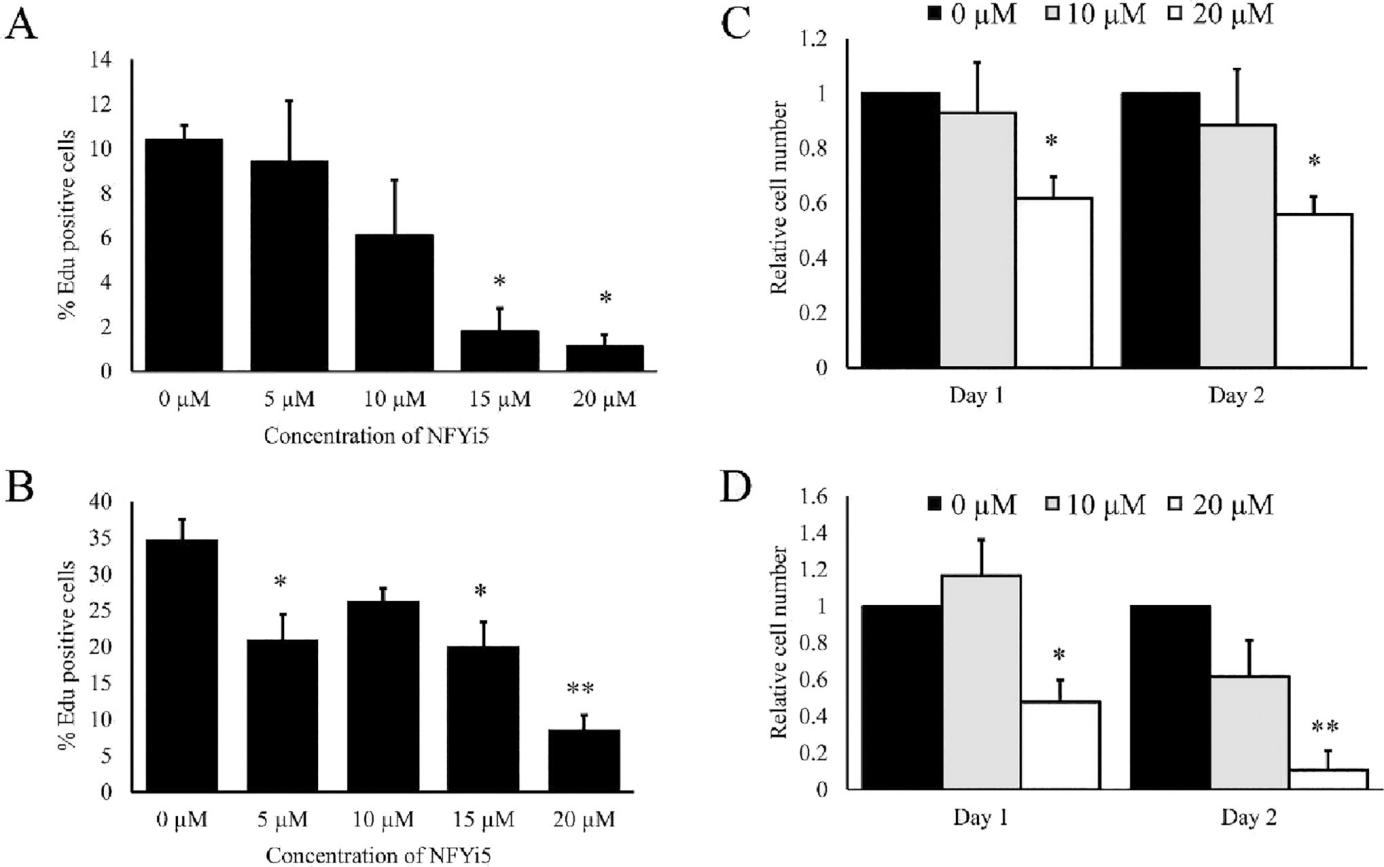
NFYi5 inhibits cell proliferation. Rat (A; *n* = 7 and C; *n* = 6) and human (B; *n* = 3 and D; *n* = 3) cardiac fibroblasts were treated with the indicated concentrations of NFYi5 for 24 h (A and B) or 24 and 48 h (C and D). Cells were labeled with 10 μM Edu for the last 4 h of the 24-h time point and Edu incorporation quantified (A and B). Total cell number was quantified after 24 and 48 h (C and D).

### NFYi5 Inhibits NF-Y DNA Binding

We performed electromobility shift assays (EMSA) to gain insight into the mechanisms underlying the inhibitory properties of NFYi5 on NF-Y activity. We initially used nuclear extracts of cells cotransfected with an equal amount of plasmid expressing NF-YA, NF-YB, and NF-YC. Incubation of a biotinylated DNA probe containing a single CCAAT binding element with nuclear extract resulted in a strong band with reduced mobility (Figure 10A). Importantly, this shifted band was still present after the addition of a 20-fold molar excess of a scrambled nonbiotinylated DNA oligo, but was completely absent when a molar excess of unlabeled probe containing a CCAAT element was added. This indicates that the shifted band represents a CCAAT-sequence-specific protein:DNA complex. We next tested whether the addition of NFYi5 to the binding reaction would affect complex formation. Importantly, NFYi5 resulted in a modest (35%) inhibition of this complex, indicating reduced NF-Y binding to the CCAAT sequence (Figure 10A). To confirm this finding, we performed EMSA using purified recombinant NF-Y protein (NF-YA, NF-YB, and NF-YC). Addition of recombinant NF-Y protein (∼100 ng) resulted in a strong band of reduced electrophoretic mobility, representing the NF-Y:DNA complex. The addition of NFYi5 significantly reduced the intensity of this complex, again indicating that NFYi5 inhibits the ability of NF-Y to bind to CCAAT-containing DNA (Figure 10B,C). NFYi5 was used at a higher (40 μM) concentration in these EMSA experiments compared to our cell-based assays, due to the relatively high levels (∼100 ng) of recombinant NF-Y protein present in the binding reactions.

**Figure 10:**
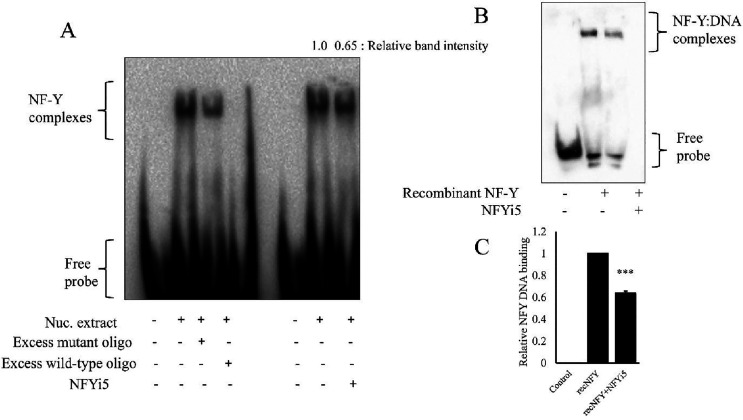
NFYi5 reduces the level of binding of NF-Y to DNA containing CCAAT. EMSA analysis of the rat cardiac fibroblast nuclear extract (A) and recombinant human NF-Y protein (B, C). Binding reactions were coincubated with a 20-fold molar excess of unlabeled oligo containing either a mutated or wild-type CCAAT motif or with 40 μM of NFYi5 (A). Numbers above lanes indicates relative optical density of NF-Y:DNA complexes. EMSA analysis of recombinant human NF-Y heterotrimer incubated in the presence of absence of 40 μM NFYi5 (B). Densitometric analysis of recombinant NF-Y:DNA complexes (C; *n* = 3).

### NFYi5 Physically Interacts with NF-YB/NF-YC

To further assess the interaction between NFYi5 and NF-Y, water-LOGSY (Water–Ligand Observation with Gradient Spectroscopy)^33^ and STD (Saturation Transfer Difference)^34^ NMR were used. In the STD experiment, the protein is selectively irradiated. This irradiation is then transferred to the binding ligand via the nuclear Overhauser effect (NOE) and detected. In the presence of NF-Y, NFYi5 gives strong STD signals in the STD NMR spectrum, indicating that this compound interacts with the NF-Y. Other small molecules, such as glycerol from the protein purification and DMSO, that do not bind or that bind weakly, are not observed in the spectrum (Figure 11). This observation is further supported by the complementary water-LOGSY experiment. In this experiment, magnetization is transferred from excited water molecules to small molecules via NOE. Binding ligands interact with water that is bound to the protein, while nonbinders only “see” the solvent water. The NOE effect from bound water has the opposite sign from the one from free water. As a result, the signal from binding fragments is opposite to the signals from nonbinders. In the presence of NF-Y, NFYi5 gives negative signals, whereas noninteracting small molecules such as glycerol or DMSO give positive peaks (Figure 12). NFYi5 was used at a higher (500 μM) concentration than in our cell-based assays (1–20 μM) due to the technical requirements of this analysis, where a large molar excess of ligand to protein is needed. Analysis of NMR spectra of 10 mM NFYi5 shows no evidence of compound aggregation at higher concentrations (Supplement Figure 4).

**Figure 11:**
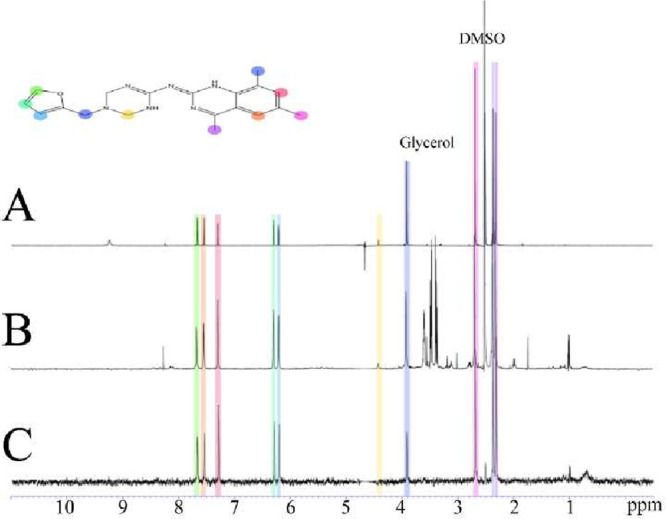
STD NMR experiment showing the binding of NFYi5 with NF-Y**.** (A) Proton spectrum of 1 mM NFYi5 in PBS, (B) STD reference, and (C) STD difference spectra of 500 μM NFYi5 in the presence of 10 μM NFY. Spectra were recorded at 20 °C at 600 MHz. The molecular structure of NFYi5 is shown with the peak assignments colored for clarity. Impurities such as glycerol from the protein purification are not observed in the STD spectrum, whereas the NFYi5 signals are present, indicating an interaction.

**Figure 12:**
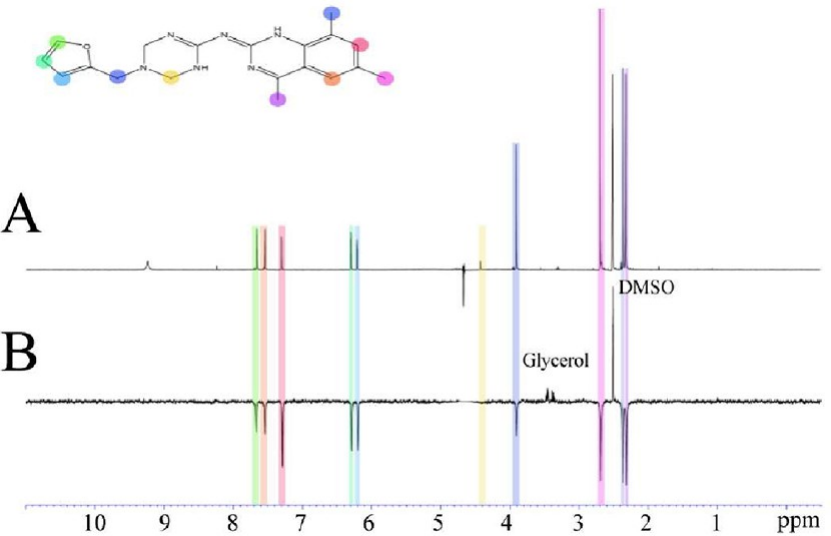
WaterLOGSY NMR experiment showing the binding of NFYi5 with NF-Y**.** (A) Proton spectrum of 1 mM NFYi5 in PBS. (B) WaterLOGSY spectrum of 500 μM NFYi5 in the presence of 10 μM NF-Y protein. Spectra were recorded at 20 °C at 600 MHz. The molecular structure of NFYi5 is shown with the peak assignments colored for clarity. Impurities such as glycerol from the protein purification and DMSO that do not bind to NF-Y show positive peaks, whereas NF5iY gives negative peaks indicating an interaction.

These NMR experiments can also provide key information about the solvent accessibility of the ligand protons. STD-based epitope mapping, in particular, can be used to distinguish ligand protons buried in the protein structure from protons exposed toward the solvent.^35^ The STD NMR data reveal that the ligand NFYi5 exhibits saturation transfer responses across the entire molecule, consistent with a docking pose in which the ligand adopts a planar orientation within the NF-Y binding groove.

### NFYi5 Induces Ubiquitin-Independent NF-YA Degradation

As these experiments indicated that NFYi5 only resulted in a partial inhibition of NF-Y DNA binding, we asked if additional mechanisms might be involved in mediating the inhibitory effects of NFYi5 on NF-Y activity. As our *in silico* molecular dynamics analysis of NF-YA indicated that NFYi5 reduced the dynamic flexibility of the NF-YA linker region (residues ∼250–270) (Figure 6), which encompasses a cluster of lysine residues implicated in NF-YA ubiquitination and turnover,^14^ we analyzed total NF-YA protein levels in cells treated with NFYi5 by Western blotting. This demonstrated a significant decrease in NF-YA protein levels (Figure 13A). Interestingly, we did not observe a reduction in NF-YA mRNA levels (data not shown), suggesting that NFYi5 modulates NF-YA protein levels at a post transcriptional level. We therefore measured the effect of NFYi5 on NF-YA protein levels in the presence of cycloheximide to block protein translation. Incubation of cells with cycloheximide alone for 8 h resulted in a small but significant reduction in NF-YA protein levels, due to the natural turnover of the protein. Coincubation with cycloheximide together with NFYi5 resulted in a larger reduction in NF-YA levels than cycloheximide alone (Figure 13B,C), indicating that the NF-YA protein is degraded at an increased rate in the presence of NFYi5. We calculated that NFYi5 reduces the half-life of NF-YA protein from 16.5 ± 1.5 h in control cells to 8.5 ± 0.7 h (*p* = 0.0433) in NFYi5-treated cells (Figure 13B–D).

**Figure 13:**
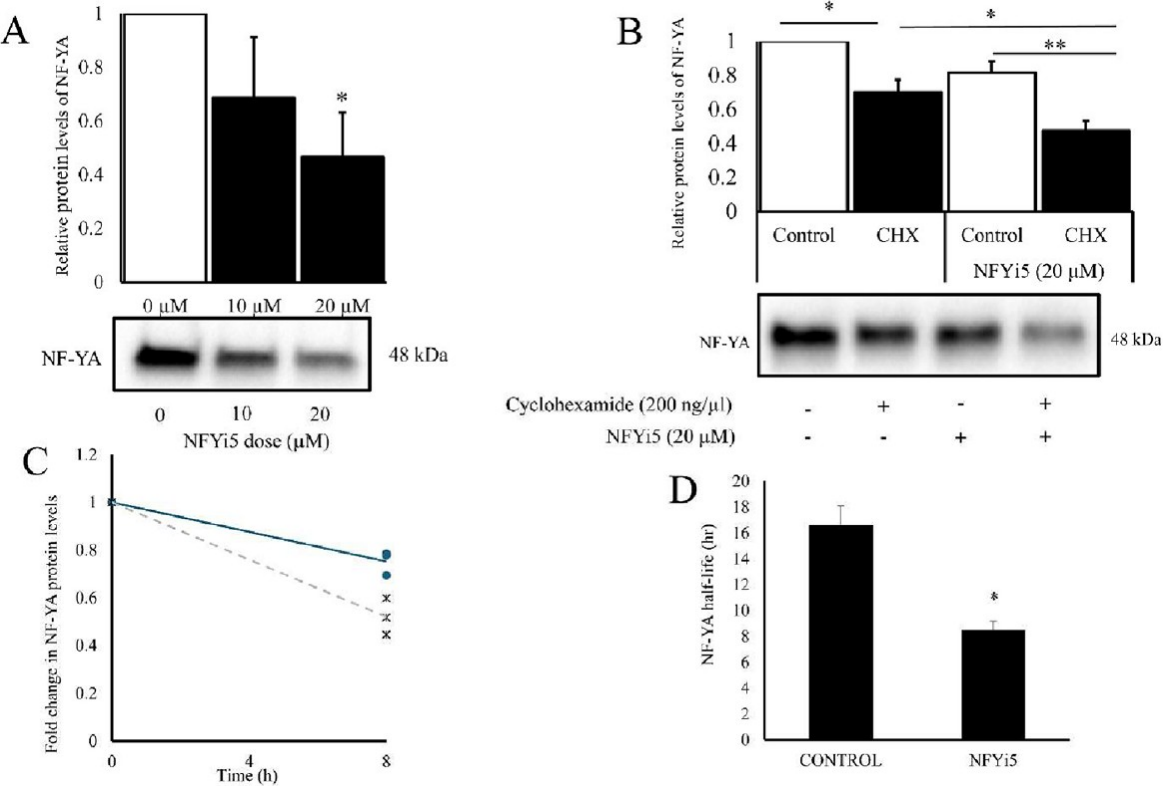
NFYi5 reduces the half-life of the NF-YA protein. Rat cardiac fibroblasts were treated with the indicated concentrations of NFYi5 and total cell lysates analyzed for NF-YA protein levels, by Western blotting, 18 h later (A; *n* = 4). Cells were pretreated with 20 μM NFYi5 for 18 h before being coincubated with 20 μM NFYi5 and cyclohexamide, to block new protein synthesis, for 8 h. NF-YA protein levels were quantified by Western blotting (B; *n* = 4). Densitometric analysis of NF-YA levels in cells coincubated with 20 μM NFYi5 and cycloheximide for 8 h (C; *n* = 4). Calculated NF-YA half-life in the presence or absence of 20 μM NFYi5 (D; *n* = 4).

We next tested if NFYi5 increases the levels of ubiquitinated NF-YA. Cells were incubated with NFYi5 for 24 h, with the last 6 h in the presence of MG132 to prevent degradation of ubiquitinated NF-YA. Ubiquitinated proteins were affinity-purified using ubiquitin affinity beads and analyzed for NF-YA by Western blotting. Although we detected NF-YA protein in the ubiquitin affinity isolated proteins, indicating a basal ubiquitination of NF-YA protein (Figure 14A,B), we did not detect more ubiquitinated-NF-YA in cells treated with NFYi5. This indicates that NFYi5 promotes NF-YA-degradation independently of increased ubiquitination. To test this further, we generated a mutant NF-YA (FLAG-NF-YA_6KtoR;_ Figure 14C) in which the six lysine residues previously demonstrated to be ubiquitinated in NF-YA and responsible for NF-YA protein turnover,^14^ and importantly located in the linker region that overlies the NFYi5 binding pocket, to arginine residues. We hypothesized that if NFYi5-mediated degradation of NF-YA was dependent on lysine ubiquitinoylation, then the FLAG-WT-NF-YA and the FLAG-NF-YA_6KtoR_ mutant would not be destabilized by NFYi5. However, we found that both the wild type and the lysine-mutated proteins were destabilized after treatment of cells with NFYi5 (Figure 14D–G). Exogenous FLAG-WT-NF-YA was found to have a half-life of 8.8 ± 0.8 h, which was decreased to 6.2 ± 0.4 h by NFYi5, whereas the FLAG-NF-YA_6KtoR_ mutant had a half-life of 8.1 ± 0.4 h, which was decreased to 5.7 ± 0.1 h by NFYi5 (Figure 14G).

**Figure 14:**
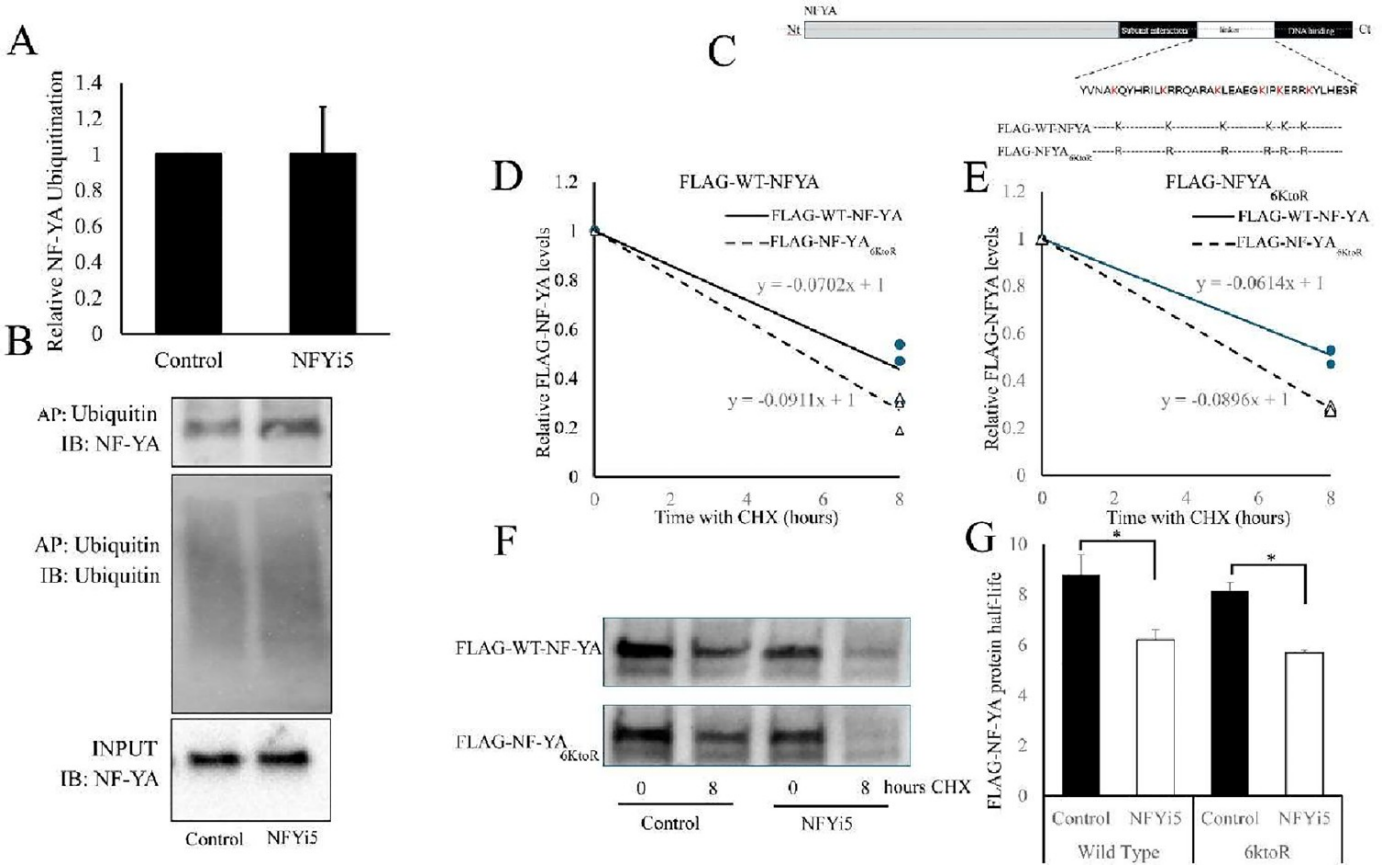
NFYi5-induced ubiquitin-independent destabilization of NF-YA. Cells were treated with 20 μM NFYi5 for 24 h with the last 6 h in the presence of 10 μM MG132. Ubiquitinated proteins were affinity purified and analyzed for NF-YA protein by Western blotting (A and B; *n* = 3). Cells were transiently transfected with plasmids expressing FLAG-WT-NF-YA or FLAG-NF-YA_6KtoR_ (C). Cells were treated with 20 μM NFYi5 for 18 h, as indicated. Cells were lysed for time point 0 or treated with 200 μg/mL cycloheximide (CHX) for a further 8 h. Levels of FLAG-NF-YA protein was quantified by Western blotting (F; *n* = 3). Protein half-life (G) was calculated from the slope of the line in the graphs (D and E). Effects of NFYi5 treatments on FLAG-WT-NF-YA or FLAG-NF-YA_6KtoR_ half-life (G; *n* = 3).

## Discussion and Conclusions

In this study, we identified and characterized a small molecule, named NFYi5, that is able to reduce the activity of the transcription factor NF-Y. Using an *in-silico* docking strategy targeting a pocket on NF-YB and screening a library of over 8 million drug-like molecules, we discovered a compound (NFYi5) that suppresses NF-Y-dependent transcription, without impairing global transcriptional activity or cell viability. NFYi5 inhibited the activity of an NF-Y-dependent reporter gene, reduced expression of canonical NF-Y target genes, inhibited proliferation of both human and rat cardiac fibroblasts, inhibited NF-Y–DNA binding, and accelerated degradation of the NF-YA subunit. Together, these findings provide proof-of-concept that NF-Y is pharmacologically tractable and establish NFYi5 as a potential lead compound for therapeutic development.

NF-Y is a well characterized regulator of cell proliferation, differentiation, and metabolism^6,8,16,17^ with NF-Y binding elements being widespread and present in almost 30% of human gene promoters.^36^ NF-Y subunits are also widely expressed, suggesting that NF-Y plays a central role in normal cellular homeostasis.^37–40^ However, specific pathology-related functions of NF-Y have also been reported. NF-YA is strongly expressed in many epithelial cancers such as breast, colon, renal, and hepatic,^9,18,41^ with expression levels associated with worse prognosis in gastric cancer,^42^ cervical cancer,^43^ and liver cancer.^44^ NF-Y has also been implicated in tissue fibrosis due to its activation by pro-fibrotic TGF-β and mechanical stimulation, and its role in promoting collagen expression and fibroblast proliferation.^3,8^ We recently demonstrated that a pathologically stiff ECM enhances NF-Y activity and promotes fibroblast proliferation, which also favors tissue fibrosis.^8^ NF-Y has been implicated in the response to vascular injury, given that NF-YA is upregulated by angioplasty induced vascular injury and local NF-Y inhibition reduces vascular remodelling.^10^ Consistent with this, NF-Y regulates many proliferative and metabolic genes that are upregulated after vascular injury and in cancer. Furthermore, CCAAT motifs are often found to be enriched in the promoters of these genes.^9,45^ These data demonstrate that NF-Y activity responds to diverse pathophysiological signals and that aberrant NF-Y activity is involved in promoting disease progression. Based on these observations, efforts have been made to inhibit NF-Y activity as a therapeutic strategy. However, targeted pharmacological inhibition of NF-Y has remained an unmet challenge.

Previous research has identified various compounds that inhibit NF-Y DNA binding and transcriptional activity. Several plant-derived compounds, including Genistein,^46^ Quercetin,^47^ and Curcumin,^48^ have been shown to block binding of NF-Y to CCAAT elements in the promoters of NF-Y-target genes. The anticancer drug ET-743 (Yondelis) was shown to inhibit the binding of several transcription factors, including NF-Y, to DNA in vitro,^49^ although later studies did not demonstrate any effect on NF-Y DNA binding in vivo.^50^ HMN-176, an active metabolite of the synthetic antitumor agent HMN-214, inhibits NF-Y DNA binding and NF-Y activity.^51^ An in silico docking study of a library of 1280 compounds identified suramin as being able to bind to the NF-Y HFD and prevent DNA binding.^20^ However, the mechanism of action of these compounds is unclear, and they are each known to have numerous targets, other than NF-Y. In an attempt to design a more selective NF-Y antagonist, Jeganathan et al. employed a structure-based design of a peptide that antagonizes recruitment of NF-YA to the NF-YB/NF-YC dimer, thus blocking formation of the functional heterotrimer.^52^ This peptide antagonist effectively blocked NF-Y DNA binding in EMSA but has not yet been shown to inhibit NF-Y synthesis in living cells. Our approach was to identify a pocket on the NF-YB/NF-YC dimer that was likely to be functionally important for NF-Y DNA binding and/or binding of NF-YA to the NF-YB/NF-YC dimer. While we demonstrate inhibition of NF-Y DNA binding (via EMSA) and activity by NFYi5, our live cell experiments show that treatment of cells with this compound is associated with suppression of NF-Y-dependent reporter activity, without negatively effecting other transcription factor-driven reporters, e.g., NF-kB or the activity of NF-Y-independent natural promoters (e.g., UBC and CMV). Furthermore, we show that NFYi5 represses the mRNA levels of previously characterized NF-Y target genes without effecting levels of NF-Y independent housekeeping genes, suggesting at least some degree of selectivity for NF-Y. A comprehensive off-target profiling remains to be conducted. The ability of NFYi5 to inhibit NF-Y:DNA binding in vitro and decrease canonical NF-Y target gene expression in cells is consistent with NF-Y–directed activity. However, we did not directly demonstrate the binding of the compound to NF-Y in living cells. In the absence of cellular target-engagement assays, off-target mechanisms cannot be excluded as contributors to the observed cellular effects. Future work, including CETSA/DARTS assays and pocket-focused genetic epistasis, will be required to confirm cellular target engagement.

It should be considered as a useful drug target. Mouse gene deletion studies show that global deletion of the NF-YA gene is embryonically lethal at 8.5 days postcoitum.^37^ This is consistent with studies showing that NF-Y is important in cell differentiation and development.^6^ The phenotype of mice with conditional deletion of the NF-YA gene in the liver,^40^ brain,^53^ adipose,^39^ or bone marrow^38^ has been reported to display a target-tissue degeneration phenotype, consistent with an essential role for NF-Y in tissue homeostasis and maintenance. Importantly, several of the NF-Y-inhibitory agents (e.g., suramin,^54^ genistein,^55^ quercetin,^56^ curcumin,^57^ ET-743,^58^ and HMN-214 ^59^) described above have successfully been used in man, despite the essential role of NF-Y in tissue homeostasis. Although these drugs have multiple targets and cannot be considered specific NF-Y inhibitors, they have all been shown to have NF-Y-inhibitory properties, implying that pharmacological manipulation of NF-Y activity is possible without the severe side effects that one may predict when considering the phenotype of global and conditional NF-YA-deleted mice.

Our data suggests that NFYi5 works via two apparently distinct mechanisms. Docking analysis suggests that NFYi5 binds to a pocket on NF-YB and NF-YC that normally accommodates the conserved NF-YA R266 residue. Our original hypothesis proposed that docking of the NF-YA Arginine-266 side chain into the pocket on NF-YB would play a role in determining optimal positioning of the DNA binding domain. However, we found that an NF-YA R266A mutant showed no reduction in the ability to promote NF-YA reporter gene activity (data not shown). Our *in-silico* modeling indicates that NF-YA is still able to bind to the NF-YB:NF-YC dimer in the presence of NFYi5, indicating that disruption of NF-YA recruitment to the NF-YB/NF-YC dimer does not account for the reduction in DNA binding. The NFYi5 docking pose places NFYi5 near the DNA helix, suggesting that interactions of NFYi5 with DNA could contribute toward inhibition of DNA binding. However, our analysis did not predict any direct interaction between NFYi5 and the DNA helix. Modeling the NF-Y heterotrimer with NFYi5 bound predicted a marked reduction in the flexibility of the NF-YA chain that links the C-terminal DNA-binding helix to the subunit interaction helix of the NF-YA protein. In addition to inhibition of DNA binding, we also demonstrated that NFYi5 results in a destabilization of NF-YA protein, reducing its half-life from 16.5 ± 1.5 to 8.5 ± 0.7 h. Our in silico modeling indicates that binding of NFYi5 induces a change in the conformation of the NF-YA chain that links the NF-YA N-terminal α helix to the C-terminal DNA binding helix. This reduction in chain flexibility is likely to impact the DNA-binding helix and may be responsible for the observed decrease in DNA binding. Importantly, this region also contains six lysine residues previously implicated as targets for ubiquitination and degradation of NF-YA.^14^ Although we were able to detect basal ubiquitination of the NF-YA protein, we did not detect increased levels of NF-YA ubiquitination after treatment with NFYi5. This implies that NFYi5 destabilizes the NF-YA protein independently of increased ubiquitination. Consistent with this, we also found that NFYi5 was as effective at destabilizing a lysine-mutated NF-YA protein as it was for wild-type NF-YA. Although most proteins are targeted for proteasomal degradation by ubiquitination, a subset have been demonstrated to undergo ubiquitin-independent proteasomal degradation.^60^ However, the ubiquitin-independent degradation of NF-YA has not been reported previously. Together, our data suggest that NFYi5 has a dual mode of action, inhibiting DNA binding and triggering the ubiquitination-independent degradation of the NF-YA subunit. The precise mechanism of NFYi5-induced NF-YA degradation is not yet clear. Future research characterizing the mechanisms underlying the ubiquitin-independent degradation of NF-YA is now required. Identification of the NF-YA interactome may help identify any interacting ubiquilin proteins, which have been implicated in mediating ubiquitin-independent protein degradation.^60^ Additionally, experiments to determine if ubiquitin-independent regulation of NF-YA occurs in response to physiologic cell signaling or if it is a unique response to NFYi5 are needed.

In summary, this study identifies and characterizes that a small molecule can modulate NF-Y–associated cellular readouts; forthcoming cellular target-engagement and genetic validation studies will determine whether NFYi5 directly binds NF-Y in cells. NFYi5, identified through structure-guided virtual screening, appears to impair NF-Y activity by disrupting DNA binding and destabilizing NF-YA, leading to potent antimitogenic effects in fibroblasts. These findings open new avenues for targeting NF-Y in diseases such as fibrosis and cancer and lay the foundation for the development of optimized NF-Y inhibitors with improved drug-like properties.

## Experimental Section

### Reagents

All chemicals were obtained from Merck unless otherwise stated. Mouse monoclonal antibody against NF-YA was from Santa Cruz. Antibody to GAPDH (MAB374) was from Merck Millipore. Antibody against Histone-H3 was from Cell Signaling Technologies. Antibody to NF-YA (sc-17753) was from Santa Cruz. Candidate NF-Y inhibitor compounds were purchased from Mcule, Inc. (Palo Alto, USA) at >95% purity. Purity of tested compounds was validated by HPLC and analysis of the NMR proton spectra (Supplement Table 2).

### *In Silico* Molecular Docking

*In silico* molecular docking was performed using the Bristol University Docking Engine (BUDE)^21^ to dock conformers generated from the ZINC database^22^ into the pocket on NF-YB that is normally occupied by the side chain of Arginine-266 of NF-YA (4AWL.pdb^13^). Briefly, the BUDE search area was defined as a 15 × 15 × 15 Å^3^ grid centered on the zeta carbon atom of the NF-YA Arginine-266 residue (Supplement Figure 1). Only NF-YB and NF-YC atoms within 20 Å of this carbon atom were included in the docking analysis. A library of > eight million compounds, obtained from the clean, druglike subset of the ZINC8 database, was used for docking studies. Multiple conformers (approximately 20 per compound) of these compounds were generated using Confort^23^ (Certara Inc.), resulting in a library of approximately 160 million distinct structures that was docked into the NF-YB/NF-YC pocket that interacts with the NF-YA R266 side chain. Each conformer was docked using 20,000 randomly generated “poses” within the search space, and the free energy of binding between the conformer and NF-YB/NF-YC was calculated. The 1000 poses with the lowest energies were selected and randomly “mutated” with *X*, *Y*, and *Z* axis translations and rotations to generate a new generation of 20,000 poses. Ten generations of this docking algorithm were performed, resulting in an optimized docking pose for each conformer and a list of all 160 million conformers ranked by the predicted free energy of binding. The top-scoring compound conformers with the lowest binding energies were shortlisted. These compounds were manually curated for chemical diversity, and 7 were selected for testing. Shortlisted hits were screened for pan assay interference compounds (PAINS) using the online PAINS filters at http://zinc15.docking.org/patterns/home/ and http://www.cbligand.org/PAINS/ (Supplement Table 2). Hit compounds passed both filters. NFYi5 was not tested for PAINS liability beyond *in silico* screening. Possible aggregation of NFYi5 at high concentrations was assessed by analysis of the NMR spectra of a 10 mM solution.

### Molecular Dynamics Simulations

Simulations were performed with GROMACS (v2024.4)^24^ on systems prepared by CHARMM-GUI.^25^ Structures were prepared in CHARMM-GUI and solvated in periodic boxes with at least 1.2 nm of solvent padding. The solution contained 0.15 M NaCl and was charge neutral. Energy minimization used the steepest descent until the maximum force fell below 1000 kJ mol^–1^ nm^–1^. Electrostatics were treated with particle-mesh Ewald using a 1.2 nm real-space cutoff; Lennard-Jones interactions employed a force-switch from 1.0 to 1.2 nm within the Verlet scheme, with neighbor lists updated every 20 steps. All X–H bonds were constrained with LINCS, and standard positional restraints were applied during preparation. Equilibration followed the CHARMM-GUI protocol: an NVT phase with velocity-rescale temperature coupling at 303.15 K, followed by NPT relaxation with an isotropic C-rescale barostat at 1 bar (water compressibility of 4.5 × 10^–5^ bar^–1^). Production simulations used leapfrog integration with a 2 fs time step, the same nonbonded settings, and separate temperature coupling for solute and solvent (velocity-rescale, 1 ps relaxation). Pressure was maintained at 1 bar with C-rescale (5 ps relaxation). Center-of-mass motion was removed every 100 steps. Compressed coordinates were written every 100 ps, and energies and logs every 2 ps. MD results were redocked with GNINA (v1.3).^26^

### Trajectory and Energetics Analysis

All trajectories were postprocessed in GROMACS to remove periodic artifacts and yield continuous, whole-molecule coordinates and were visualized via PyMOL.^27^ PyLipID^28^ was applied to locate and quantify primary binding sites. Binding energetics were estimated with MM/PBSA using gmx mmpbasa^29^ and per-residue decomposition.

### Preparation of Recombinant NF-Y Protein

The full-length coding sequence of human NF-YA (NM_002505.5) was cloned into the prokaryotic expression vector pGEX-6T-1 to produce a recombinant protein with a N-terminal GST-tag. The DNA-binding domains of human NF-YB (residues 52–140; NP_001401447.1) and human NF-YC (residues 35–110; NP_001136062.1) were expressed as a single polypeptide chain by joining the C-terminus of NF-YB to the N-terminus of NF-YC with a short linker sequence (GGSLEVLFQGPGGST). Recombinant GST-tagged human NF-YA and NF-YB/NF-YC were expressed in the BL21 strain of *E. coli*. Protein expression was induced with 0.25 mM IPTG at 22 °C for 3 h. Soluble protein was extracted using CellLytic B lysis buffer (Merck), followed by a second extraction of the insoluble material using PBS/2 M Urea. GST-tagged proteins were batch-purified using glutathione-agarose beads. Proteins were eluted in 20 mM reduced glutathione.

### Cardiac Fibroblast Culture

Sprague–Dawley rats were killed by inhalation of CO_2_ in accordance with Schedule 1 of the U.K. Animals (Scientific Procedures) Act 1986 and Directive 2010/63/EU of the European Parliament and with the approval of the University of Bristol. Hearts were removed and flushed with PBS to remove residual blood before being chopped into 2 mm^2^ pieces. Pieces of myocardium were digested with 2 mg/mL collagenase (Worthington Biochemical Corporation) overnight. The cell suspension was pelleted and resuspended in Advanced DMEM/F12 supplemented with 10% fetal bovine serum, 100 U/mL penicillin/streptomycin, and 2.5 mM l-glutamine. Cells were allowed to adhere to tissue culture plastic for 72 h, and nonadherent myocytes were washed away. Cultures were expanded by serial passage and used in experiments between passages 4 and 10. Human cardiac fibroblasts were isolated from pieces of human atrial appendage (Ethical approval REC: 2/SW/0128) that were digested with 1 mg/mL collagenase I (Worthington) in HEPES-buffered DMEM for 4 h at 37 °C. Fibroblasts were adhered to tissue culture flasks overnight, and nonadherent myocytes were discarded.

### Reporter Gene Assays

NF-Y reporter plasmid, consisting of a secreted nanolucifase reporter gene under the regulation of a synthetic promoter containing five copies of a consensus CCAAT binding element has been previously described.^8^ NanoLUC reporter plasmids containing a synthetic promoter containing five copies of a consensus NF-kB element or a CMV promoter were obtained from Promega. A NanoLuc reporter plasmid containing 1212 bp of the human UBC promoter was obtained from Addgene (Plasmid #113450)**.** Rat cardiac fibroblasts were transiently transfected with 5 μg of reporter plasmid by electroporation using a nucleofector 1.5 (program A-024) and allowed to recover overnight. Cells were then treated with indicated concentrations of compound for 18 h. Cells were washed and fresh media added, followed by an incubation for a further 6 h to collect secreted NLUC enzyme or cells lysed using Cell Culture Lysis buffer (Promega) for nonsecreted reporters. NLUC activity was assayed by using the Promega NanoGlo assay kit.

### RNA Extraction, Quantitative Real-Time PCR

Total RNA was prepared using Qiagen RNeasy mini columns according to the manufacturer’s instructions. RNA was quantified by reading the OD_260_ values using a nanodrop spectrophotometer. For qPCR, equal amounts of RNA were converted to cDNA using a Qiagen QuantiTect first-strand cDNA synthesis kit with random hexamer priming. Quantitative Real-Time PCR was performed using KappaFAST SYBR Green using a Qiagen Roto-Gene Q PCR machine (15’@95 °C;15’@62 °C;5′@72 °C). Primers sequences are described in Supplement Table 1. Data were normalized to the total amount of RNA, unless otherwise indicated.

### Western Blotting

Total cell lysates were prepared in 1× reducing Laemmli sample buffer (2% SDS, 10 glycerol, 50 mM Tris, pH 6.8, 2.5% β-mercaptoethanol, and 0.002% bromophenol blue). Proteins were denatured by heating to 95 °C for 5 min before electrophoresis using Bio-Rad 4–15% polyacrylamide mini-TGX gels in a Mini-Protean II electrophoresis apparatus. Proteins were transferred to TransBlot PVDF membrane (BioRad) using a semidry Turbo blotter system (Bio-Rad). Membranes were blocked for 1 h at room temperature in 5% low-fat milk powder in Tris buffered saline (50 mM Tris-HCl, pH 7.6, 150 mM NaCl, 2 mM KCl) containing 0.2% Tween20 (1× TBS.T) before incubation with primary antibody overnight at 4 °C. Blots were extensively washed in 1× TBS.T before incubation with HRP-conjugated secondary antibodies (Sigma) for 1 h at room temperature. Specific proteins were detected using the Immobilon ECL reagent and a ChemiDoc-MP digital imaging system (Bio-Rad).

### Cell Proliferation Assays

Cell proliferation was quantified using EdU incorporation and counting of total cell number/mm^2^. Unless otherwise stated, all experiments were conducted using asynchronously proliferating cells cultured in Advanced DMEM/F12 supplemented with 5% fetal bovine serum, 100 U/mL penicillin/streptomycin, and 2.5 mM l-glutamine. For EdU labeling assays, cells were treated as indicated and incubated with 10 μM EdU for 4 h. Cells were fixed in 70% ethanol, and EdU incorporation was detected using the EdU-CLICK-488 assay kit (SIGMA). Nuclei were counterstained with DAPI, and EdU-positive and total nuclei numbers were manually counted using ImageJ software. For total cell numbers, cells were fixed in 70% ethanol, −24, −48, and −72 h post treatment, and nuclei were stained with DAPI. Nuclei were counted using Cell Profiler software and expressed as cells/mm^2^.

### DNA Binding Assays

NF-Y DNA binding was analyzed using an electrophoretic mobility shift assay. DNA binding of NF-Y was analyzed using cardiac fibroblast nuclear extracts prepared using the NE-PER nuclear and cytoplasmic extraction kit (Thermo Fisher Scientific) and purified recombinant GST-tagged NF-YA and NF-YB/NF-YC fusion (described above). DNA binding reactions contained 10 mM Tris-HCl, pH 7.6, 100 mM KCl, 10% glycerol, 1 mM EDTA, 2 mM MgCl_2_, 2 mM DTT, 50 ng/ul poly­[dI-dC], 1 nM 5′ biotinylated DNA oligo. Binding reactions were supplemented with 50 μM NFYi5, as indicated. Reactions were incubated at room temperature for 30 min before electrophoresis on a 6% polyacrylamide gel in 0.5× TBE buffer pH 8.0. Following electrophoresis, gels were electroblotted onto a positively charged nylon membrane. DNA oligos were cross-linked onto the membrane by exposure to 120,000 μJ/cm^2^ of UV light. Biotinylated DNA oligos were then detected using the Lightshift chemiluminescent EMSA kit (Thermo Fisher Scientific), according to the manufacturer’s instructions.

### Ubiquitination Assays

NF-YA ubiquitination was analyzed using the SignalSeeker Ubiquitination assay (Cytoskeleton, Inc.), according to the manufacturer’s instructions. Briefly, cells were treated with 20 μM NFYi5 for 24 h, with the final 6 h in the presence of 10 μM MG132. Ubiquitinated proteins were affinity isolated from whole cell lysates using the SignalSeeker Ubiquitination kit (Cytoskeleton Inc.). Ub-affinity-isolated proteins were analyzed for NF-YA by Western blotting.

### Saturation Transfer Difference and WaterLOGSY NMR

A 10 μM stock of NF-Y protein was prepared in a phosphate buffer with 10% D_2_O (75 mM potassium phosphate and 150 mM sodium chloride at pH 7.5). The ligand was dissolved in DMSO-*d*6 to 10 mM with 0.5% formic acid-*d*2 to fully solubilize. NMR spectra were acquired with a Bruker NEO spectrometer operating at 600 MHz equipped with a 5 mm TXO cryoprobe with *z* gradients at 20 °C. Standard Bruker pulse programs with excitation sculpting were used to suppress the water peak. Both the STD and water-LOGSY experiments were recorded on the same sample with 500 μM ligand in a 3 mm tube with a relaxation delay of 2 s. The waterLOGSY experiment had a mixing time of 1.7 s while the STD had a saturation time of 2 s with the on- and off-resonance saturation frequencies set to 0.7 and −40 ppm, respectively. To assess the propensity of the ligand to aggregate in aqueous conditions, the ligand stock described above was diluted to 1 mM in PBS, and a proton was recorded.

### HPLC Analysis

Analytical HPLC analyses were conducted on an Agilent 1260 Infinity II LC–MS system equipped with a Poroshell 120 EC-C18 column (4 μm, 4.6 × 100 mm). Elution was performed using a gradient of water/acetonitrile containing 0.5% formic acid from 95:5 to 0:100 over 10 min, at a flow rate of 1.0 mL/min. UV detection was monitored at 254 nm. Representative chromatograms and integrated peak area data are provided in the Supporting Information (Supplement Figures 5–10 and Supplement Table 2). Under these analytical conditions, all compounds exhibited purities of ≥94%.

### Data and Statistical Analysis

Raw experimental data were collated and graphed using Microsoft Excel, with final figures constructed using Microsoft PowerPoint. Statistical analysis was performed using Graphpad Instat software (https://www.graphpad.com/scientific-software/instat/). Reported number of independent experiments performed using different preparations of CF. Data are presented as means ± standard error of the mean. After testing for normal distribution, data were analyzed either using one-way ANOVA with Student–Newman–Keuls post-test, or, where appropriate student’s *t-*test, as indicated.

## Supplementary Material







## Notes

Render and overexpression of NF-Y; MD analysis; and HPLC and MS analyses (PDF)
Table of qPCR primer sequences and table of compound properties (XLSX)
MD simulations to test if NFYi5 docking to NF-Y is stable over time and to refine the initial pose (MP4)

## Notes

NF-Y 3D structure used for docking studies is 4AWL.pdb.^13^

## Abbreviations

CF: cardiac fibroblasts
CMV: cytomegalovirus
ECM: extracellular matrix
EMSA: electromobility shift assay
NF-Y: nuclear transcription factor-Y
NF-YA: nuclear transcription factor-Y subunit A
NF-YB: nuclear transcription factor-Y subunit B
NF-YC: nuclear transcription factor-Y subunit C
NFYi: nuclear transcription factor-Y inhibitor
NMR: nuclear magnetic resonance
STD: saturation transfer difference
