# Supplementary material for: In Silico Discovery and Characterization of a Novel Nuclear Transcription Factor‑Y (NF-Y) Inhibitor with Antimitogenic Properties
Source: J Med Chem. 2026 Mar 20;69(7):8115–29. doi: 10.1021/acs.jmedchem.5c03508 (PMC13071875; doi:10.1021/acs.jmedchem.5c03508)
Supplement: Supplementary file 1 [file jm5c03508_si_001.pdf]

SUPPORTING INFORMATION

In silico discovery and characterisation of a novel nuclear transcription factor-Y (NF-Y) inhibitor with anti-mitogenic properties

Reza Ebrahimighaei[1], Jon Lees[1], Robin A. Corey[2], Boyi Xiao[2], Christopher Williams[3], Himali Y. Godage[3], Vealmurugan Sekar[1], Hunaid Vohra[3], Deborah Shoemark[2], Andrew Newby[1] and Mark Bond[1]*

*Corresponding author: Mark Bond (mark.bond@bris.ac.uk)

Affiliations:

1. Translational Health Sciences Bristol Medical School, University of Bristol, Bristol BS2 8HW, U.K.
2. School of Physiology, Pharmacology and Neuroscience, Biomedical Sciences Building, University of Bristol, BS8 1TD
3. Bristol Heart Institute, University Hospitals Bristol, NHS Foundation Trust, Bristol, BS1 3NU, U.K.

CONTENTS

FIGURE	TITLE	PAGE
Supplement Figure 1	Render of NF-Y (4AWL.pdb) including the 15-angstrom cube defining the BUDE molecular docking search area.	S3
Supplement Figure 2	Overexpression of NF-YA stimulates activity of a nanoluciferase reporter gene driven by a synthetic promoter containing multimerised NF-Y binding elements	S4
Supplement Figure 3	Molecular dynamic analysis hydrogen bond frequency heat map between NF-YB residues and NFYi5 atoms	S5
Supplement Figure 4	NMR proton spectrum of 10 mM NFYi5 in DMSO	S5
Supplement Figure 5	HPLC and MS analysis of NFYi2	S6
Supplement Figure 6	HPLC and MS analysis of NFYi3	S8
Supplement Figure 7	HPLC and MS analysis of NFYi4	S9
Supplement Figure 8	HPLC and MS analysis of NFYi5	S10
Supplement Figure 9	HPLC and MS analysis of NFYi6	S11
Supplement Figure 10	HPLC and MS analysis of NFYi7	S12

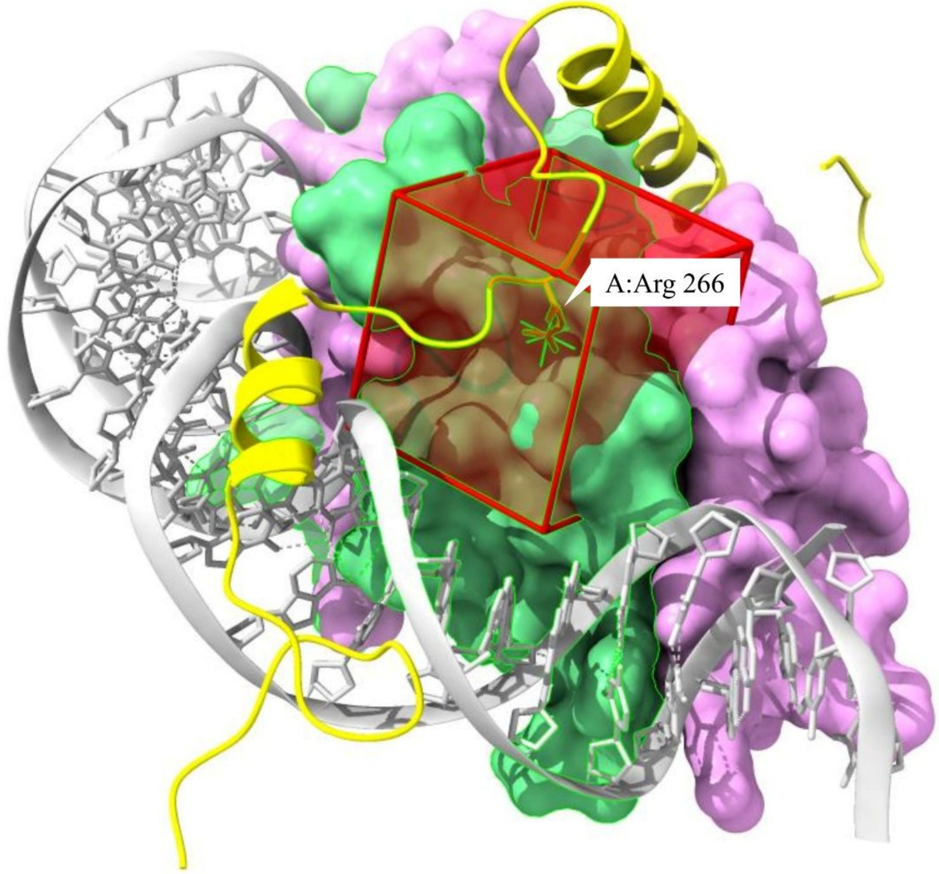

Supplement Figure 1. Render of NF-Y (4AWL.pdb) including the 15-angstrom cube defining the BUDE molecular docking search area. NF-YA in yellow with R266 side chain shown. NF-YB in green. NF-YC in pink. Red box indicated BUDE docking search area.

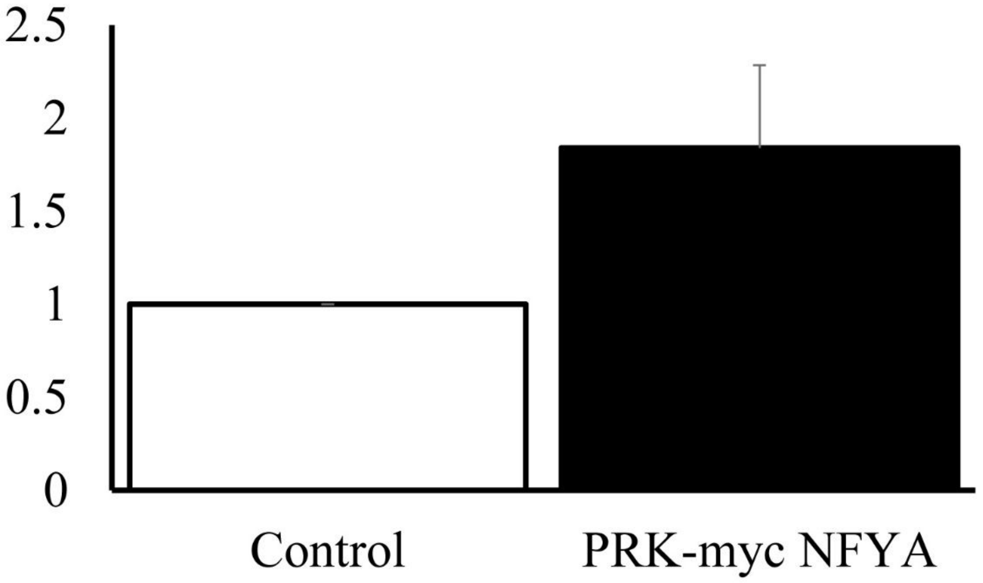

Supplement figure 2: Overexpression of NF-YA stimulates activity of a nanoluciferase reporter gene driven by a synthetic promoter containing multimerised NF-Y binding elements.

Rat fibroblasts were transiently transfected with NF-Y-NLUC reporter plasmid together with either empty expression vector or pRK-Myc-NF-YA. Nano-luciferase activity was quantified 24 hour post transfection.

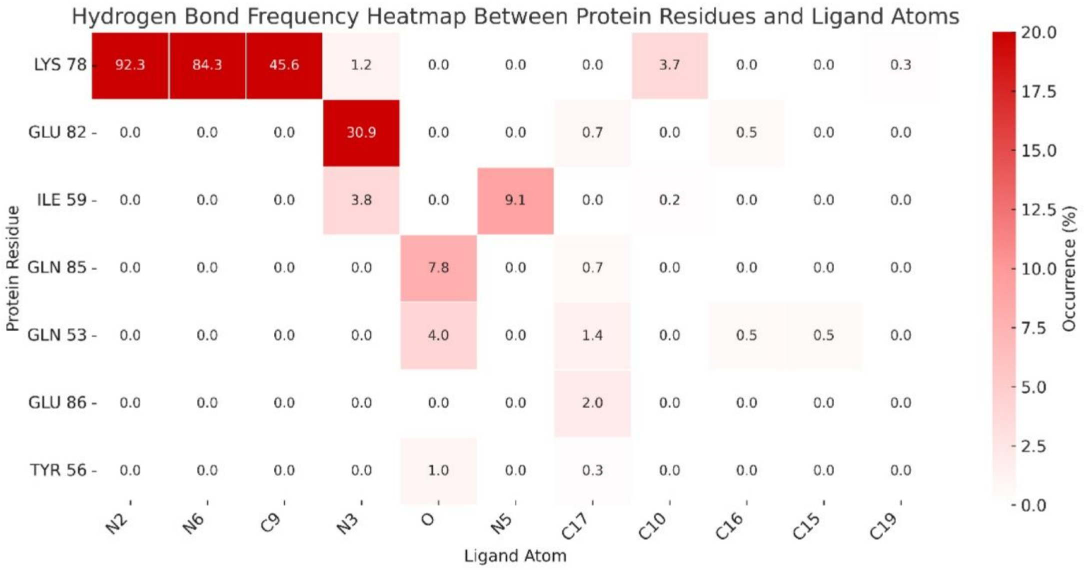

Supplement Figure 3: Molecular dynamic analysis hydrogen bond frequency heat map between NF-YB residues and NFYi5 atoms

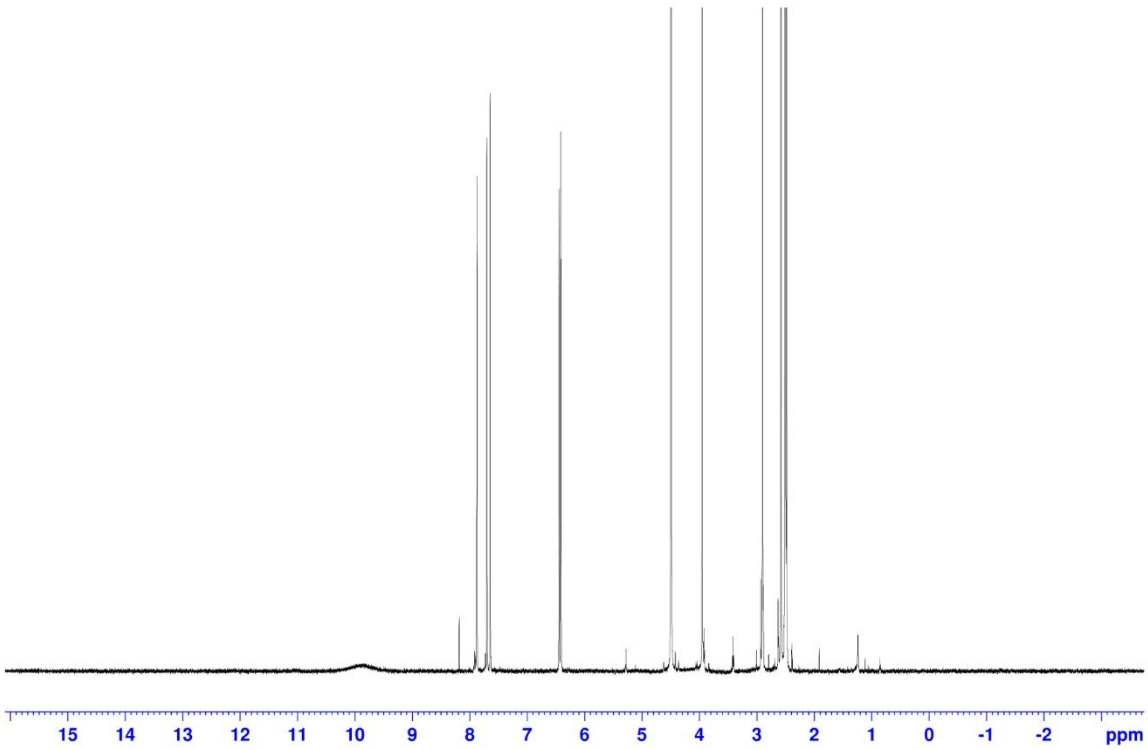

Supplement Figure 4 NMR proton spectrum of 10 mM NFYi5 in DMSO

A
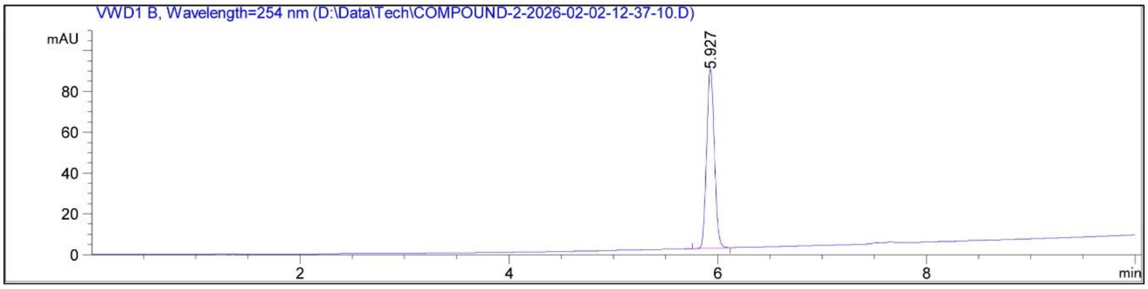

B

Peak #	RetTime [min]	Type	Width [min]	Area [mAU*s]	Height [mAU]	Area %
1	5.927	BB	0.0801	453.71442	88.04765	100.0000

C
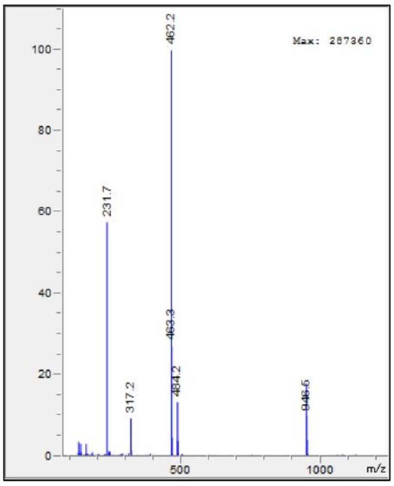

Supplement Figure 5: HPLC and MS analysis of NFYi2

HPLC trace of NFYi2 (A). Analysis table of HPLC data (B), MS spectra of NFYi2 (C)

A

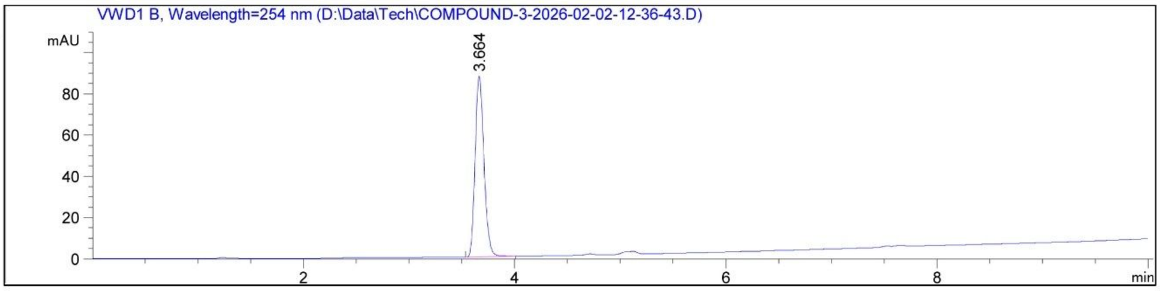

B

Peak #	RetTime [min]	Type	Width [min]	Area [mAU*s]	Height [mAU]	Area %
1	3.664	BB	0.0902	512.00482	87.61877	100.0000

C

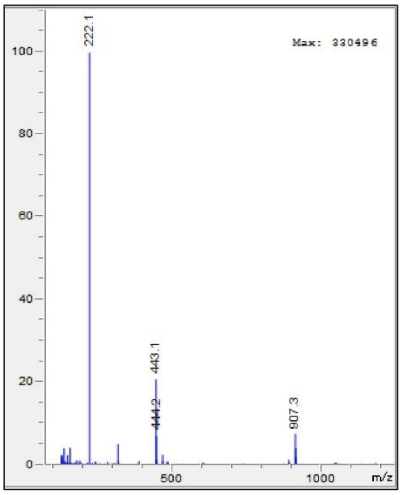

Supplement Figure 6: HPLC and MS analysis of NFYi3

HPLC trace of NFYi3 (A). Analysis table of HPLC data (B). MS spectra of NFYi3 (C).

A

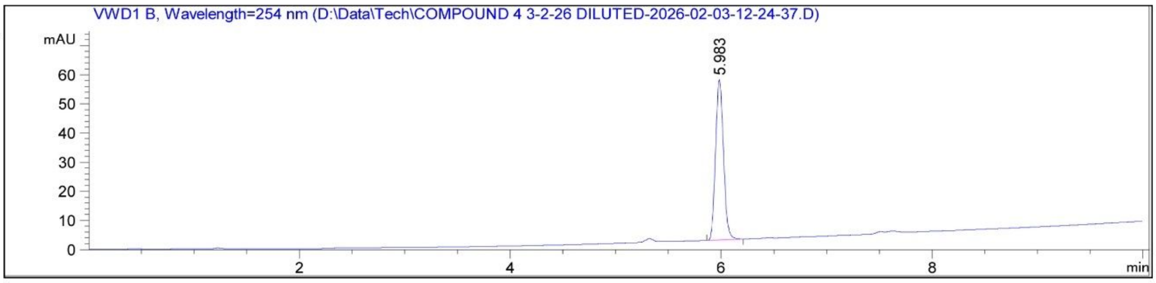

B

Peak #	RetTime [min]	Type	Width [min]	Area [mAU*s]	Height [mAU]	Area %
1	5.983	BB	0.0832	289.07162	55.10823	100.0000

C

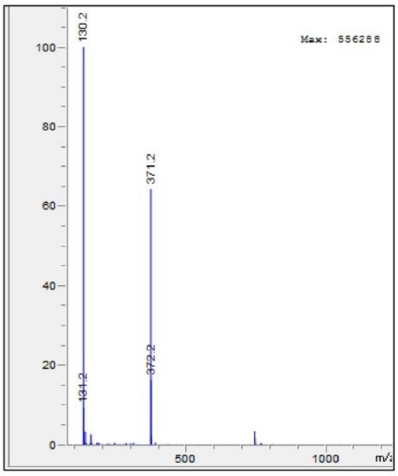

Supplement Figure 7: HPLC and MS analysis of NFYi4

HPLC trace of NFYi4 (A). Analysis table of HPLC data (B). MS spectra of NFYi4 (C).

A

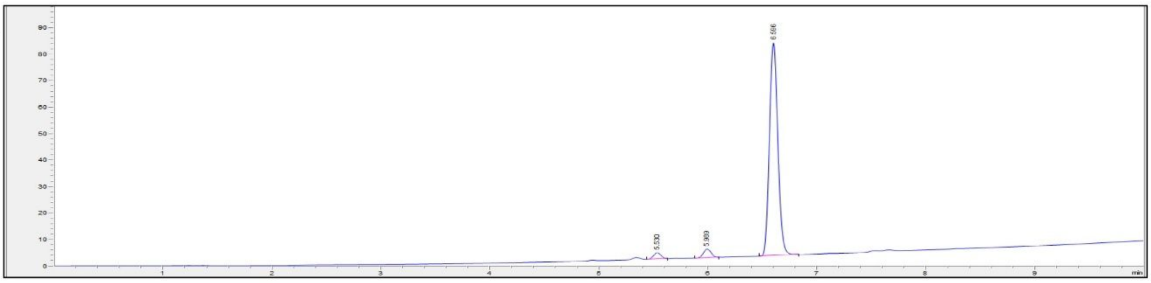

B

Peak #	RetTime [min]	Type	Width [min]	Area [mAU*s]	Height [mAU]	Area %
1	5.530	BB	0.0781	11.40754	2.37045	2.4698
2	5.989	BB	0.0794	16.01160	3.25602	3.4666
3	6.596	BB	0.0849	434.45859	80.58906	94.0635

C

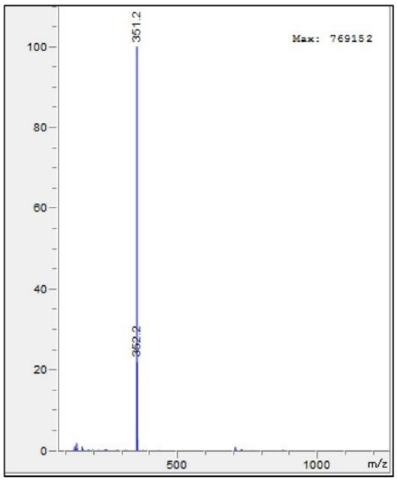

Supplement Figure 8: HPLC and MS analysis of NFYi5

HPLC trace of NFYi5 (A). Analysis table of HPLC data (B). MS spectra of NFYi5 (C).

A

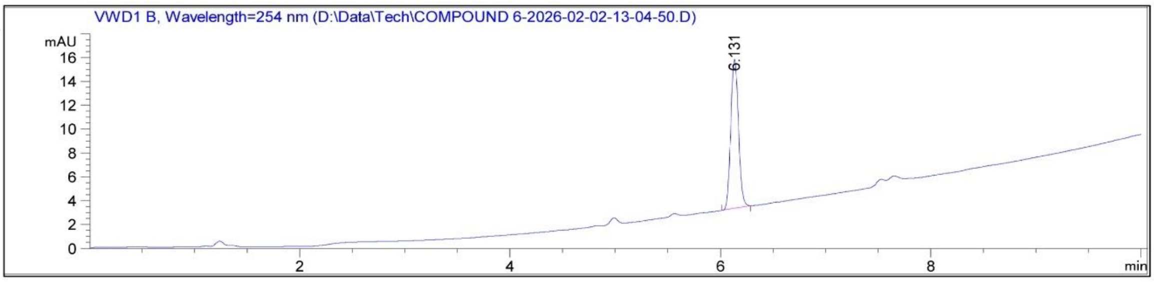

B

Peak #	RetTime [min]	Type	Width [min]	Area [mAU*s]	Height [mAU]	Area %
1	6.131	BB	0.0792	63.35761	12.48605	100.0000

C

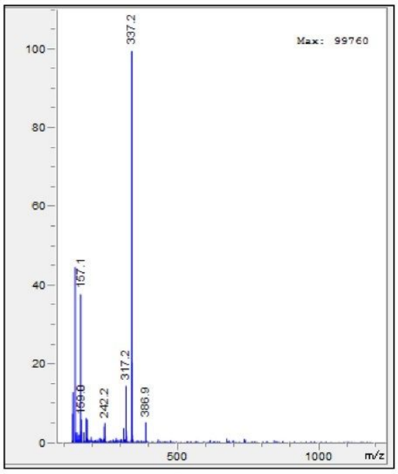

Supplement Figure 9: HPLC and MS analysis of NFYi6

HPLC trace of NFYi6 (A). Analysis table of HPLC data (B). MS spectra of NFYi6 (C).

A

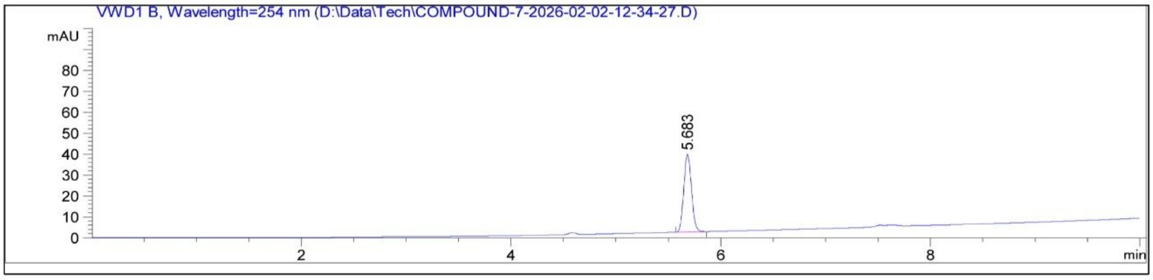

B

Peak #	RetTime [min]	Type	Width [min]	Area [mAU*s]	Height [mAU]	Area %
1	5.683	BB	0.0818	190.05534	37.07333	100.0000

C

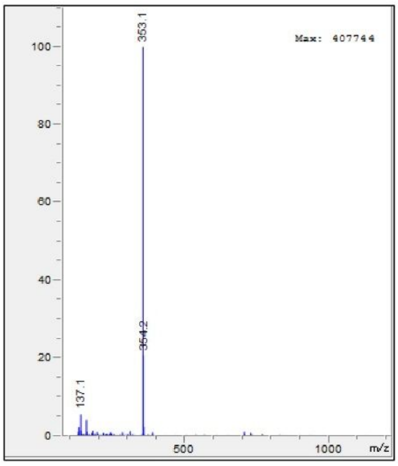

Supplement Figure 10: HPLC and MS analysis of NFYi7

HPLC trace of NFYi7 (A). Analysis table of HPLC data (B). MS spectra of NFYi7 (C).
